# Cover Crop Root Channels Promote Bacterial Adaptation to Drought in the Maize Rhizosphere

**DOI:** 10.1111/gcb.70512

**Published:** 2025-09-20

**Authors:** Debjyoti Ghosh, Yijie Shi, Iris M. Zimmermann, Katja Holzhauser, Martin von Bergen, Anne‐Kristin Kaster, Sandra Spielvogel, Michaela A. Dippold, Jochen A. Müller, Nico Jehmlich

**Affiliations:** ^1^ Department of Molecular Toxicology Helmholtz Centre for Environmental Research—UFZ GmbH Leipzig Saxony Germany; ^2^ Department of Soil Science, Institute of Plant Nutrition and Soil Science Christian‐Albrechts‐University Kiel Kiel Schleswig‐Holstein Germany; ^3^ Institute of Crop Science and Plant Breeding, Agronomy and Crop Science Christian‐Albrechts‐University Kiel Kiel Schleswig‐Holstein Germany; ^4^ Institute for Biochemistry, Faculty of Biosciences, Pharmacy and Psychology University of Leipzig Leipzig Saxony Germany; ^5^ German Centre for Integrative Biodiversity Research (iDiv) Halle‐Jena‐Leipzig Leipzig Saxony Germany; ^6^ Institute for Biological Interfaces Karlsruhe Institute of Technology Eggenstein‐Leopoldshafen Baden‐Württemberg Germany; ^7^ Department of Geosciences, Geo‐Biosphere Interactions University of Tübingen Tübingen Baden‐Württemberg Germany

**Keywords:** bacterial community, cover crop, drought, metaproteomics, root channel re‐use, soil types

## Abstract

Increasing drought frequency poses a significant threat to agricultural productivity. A promising strategy to enhance crop resilience against drought is the utilisation of root channels left by winter cover crops, which can improve access to subsoil water and nutrients for subsequent cash crops like maize ( *Zea mays* L.). The impact of drought on bacterial communities inhabiting these root channels remains largely unknown. Here, we investigated drought‐induced shifts in maize rhizosphere bacterial communities and their functional adaptation in cover crop root channels across three soil types in northern Germany (Luvisol, Podzol, and Phaeozem) using a multi‐omics approach (16S rRNA gene amplicon sequencing, qPCR, and metaproteomics). Our results reveal a preference towards bacterial *K*‐strategists under drought conditions, indicating a shift towards stress‐tolerant populations. Under drought stress, the relative abundances of *Acidobacteriota*, *Actinomycetota*, *Planctomycetota*, and *Pseudomonadota* increased, while *Chloroflexota*, *Methylomirabilota*, *Ca*. Patescibacteria, and *Verrucomicrobiota* declined. Metaproteomics analyses revealed that drought‐stressed aerobic taxa among the *Pseudomonadota* and *Verrucomicrobiota* upregulated the glyoxylate cycle, potentially enhancing carbon and energy conservation, and increased antioxidant defences (catalase–glutathione peroxidase and methionine cycle–transsulfuration pathway). These drought‐mitigating strategies were especially pronounced in root channels formed by *Brassicaceae* and *Poaceae* cover crops in the Luvisol and Podzol soils. These findings demonstrate the functional plasticity of rhizosphere bacterial communities in reused root channels in response to drought, highlighting the potential to leverage microbiome‐mediated resilience for agricultural practices.

## Graphical abstract

In this study, we examine the structural and functional changes in the bacterial communities in the rhizosphere of a summer cash crop (maize) that reuses root channels from winter cover crops to improve access to subsoil water and nutrient resources and address threats posed by the increased frequency of droughts. K‐strategists and bacteria preferring nutrient‐poor conditions increase relative to fallow during drought stress across soil types. Pseudomonadota, Spirochaetota, and Verrucomicrobiota upregulate the glyoxylate cycle and antioxidant pathways to mitigate stress and provide valuable insights about microbiome‐mediated resilient sustainable agricultural practices.

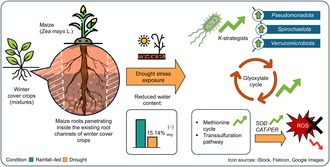

## 1 Introduction

Increasing drought frequency and intensity, driven by climate change, significantly impact crop production worldwide (Masson‐Delmotte et al. 2018; Tahasin et al. 2024; Xu et al. 2019). Globally, the total gross primary production (GPP) reduced by 28% during 1850–1999 under extreme drought conditions and is predicted to rise even further in the near future (Xu et al. 2019). Drought primarily impacts topsoil (Sanaullah et al. 2012), reducing water availability and limiting nutrient mobility and uptake (Amtmann and Blatt 2009). Enhancing a plant's access to subsoil resources offers a promising strategy to mitigate these effects and sustain crop yields (Frelih‐Larsen et al. 2018; Querejeta et al. 2021). Utilizing root channels created by previous cover crops can facilitate deeper root penetration and subsoil resource acquisition by subsequent cash crops (Ghosh et al. 2024; Huang et al. 2020; Zhou et al. 2020). These channels are recognized hotspots of microbial activity, exhibiting enhanced biogeochemical cycling compared to bulk soil (Kuzyakov and Blagodatskaya 2015). In channels with decaying roots, the microbial activities are largely driven by residual complex root exudates and rhizodeposits, while in channels with viable roots, new labile plant‐derived organic carbon mainly serves as microbial substrate (Banfield et al. 2017). Using maize as a model cash crop, we previously showed that bacterial abundances and functional diversity increase in the rhizosphere when maize roots utilize cover crop root channels compared to plants grown in unconditioned bulk soil (Ghosh et al. 2024). A notable rise in bacterial presence contributed to a higher expression of metabolic pathways after maize roots reused the root channels, possibly due to improved access to subsoil nutrients.

This increased bacterial metabolic activity in the root channels could change with distinct soil types, as different soil classifications have unique compositional profiles. The World Reference Base for Soil Resources categorized soil into different types based on the composition (Schad 2023). According to the list, Luvisols and Phaeozems are rich in high‐activity clays and dark, loamy organic matter, respectively, with high‐base status, while Podzols feature a significant sand proportion and are mostly acidic (Eugenio D'Amico et al. 2023). Soil texture (loamy, clayey, or sandy) significantly influences crop cultivation and yield by affecting root growth, water and nutrient uptake, and microbial community structure (Giuliani et al. 2024; Liu, Huang, et al. 2020). Luvisol is classified as a sandy loam (41% sand, 37% silt, 22% clay), Phaeozem is a silt loam (35% sand, 54% silt, 11% clay), and Podzol is a sandy sand (91% sand, 6% silt, 3% clay) (Grosse et al. 2021; Voelkner et al. 2019). Bacterial diversity increased in soils having higher proportions of sand, followed by loam and clay, where members of the phyla *Acidobacteriota*, *Actinomycetota*, *Bacillota*, *Bacteroidota*, *Chloroflexota*, and *Pseudomonadota* were the most dominant ones (Zheng et al. 2021). Pore size and coarseness in soils of different textures influence the abundance of these phyla (Xia et al. 2022), as well as soil fertility by changing nutrient availabilities (Cao et al. 2021). For instance, classes of *Pseudomonadota* such as *Alphaproteobacteria* showed preferences to inhabit micropores, while *Betaproteobacteria* preferred macro‐ and mesopores. Under drought conditions, the bacterial community structure may shift in response to altered resource availability affected by the drying out of the soil (Caddell et al. 2023; Metze et al. 2023). Compared to r‐strategists, *K*‐strategists can cope better with reduced nutrient supply under such conditions (Dytczak et al. 2008; Yin et al. 2022), so the propensity of an increasing relative *K*‐strategist presence is high. Given that the abundance of different bacterial phyla is influenced by pore sizes and altered nutrient supplies under changing conditions, the texture of different soil types may play a significant role in determining community structures. Whether the structure, function, and dynamics of bacterial communities inhabiting the root channels are influenced by soil texture or the cover crop root channel properties being predominant remains poorly understood (Xia et al. 2020; Xue et al. 2023). Understanding these interactions is crucial for optimizing cover cropping strategies and improving crop resilience to drought.

Under oxidative stress, dry soils lead to an increase in the volume of reactive oxygen species (ROS) produced upon the formation of cracks in the soil. ROS damage cellular components like nucleic acids, proteins, and lipids (Imlay 2013) and thus are detrimental for the survival of bacteria. Catalase (CAT), glutathione peroxidase (GPX), and superoxide dismutase (SOD) have indispensable roles in regulating the proportion of different types of ROS in a system (Ighodaro and Akinloye 2018). However, most of the research relevant to soil systems subjected to drought was focused on plants, which encouraged us to look at the communities in the root rhizospheres—one of the most active regions belowground. The structural and functional dynamics of bacterial communities in these active regions are underexplored, and the addition of cover crops further increases the complexity of the system, underscoring the need for more research.

To address the knowledge gaps, we conducted field experiments using maize as a cash crop following mixtures of winter‐grown cover crops. Drought conditions were simulated in a rainout shelter experiment. We employed 16S rRNA gene amplicon sequencing, quantitative PCR (qPCR), and metaproteomics to examine the effects of drought (D) compared to rainfall‐fed (RF) maize cultivation on the structure and function of the rhizosphere bacterial community in maize reusing pre‐existing root channels across different soil textures. We hypothesized that the bacterial microbiome of the maize rhizosphere reusing cover crop root channels is more active and better at implementing adaptive mechanisms to mitigate drought‐induced stress in a soil‐type‐specific manner. This research aims to elucidate rhizosphere microbiome dynamics and adaptations in the biochemical pathways in topsoil and subsoil with varying textures under drought conditions, ultimately informing strategies for selecting cover crops and enhancing crop resilience in water‐limited environments.

## 2 Methods

### 2.1 Crop Cultivation and Sampling Regimes

**FIGURE 1:**
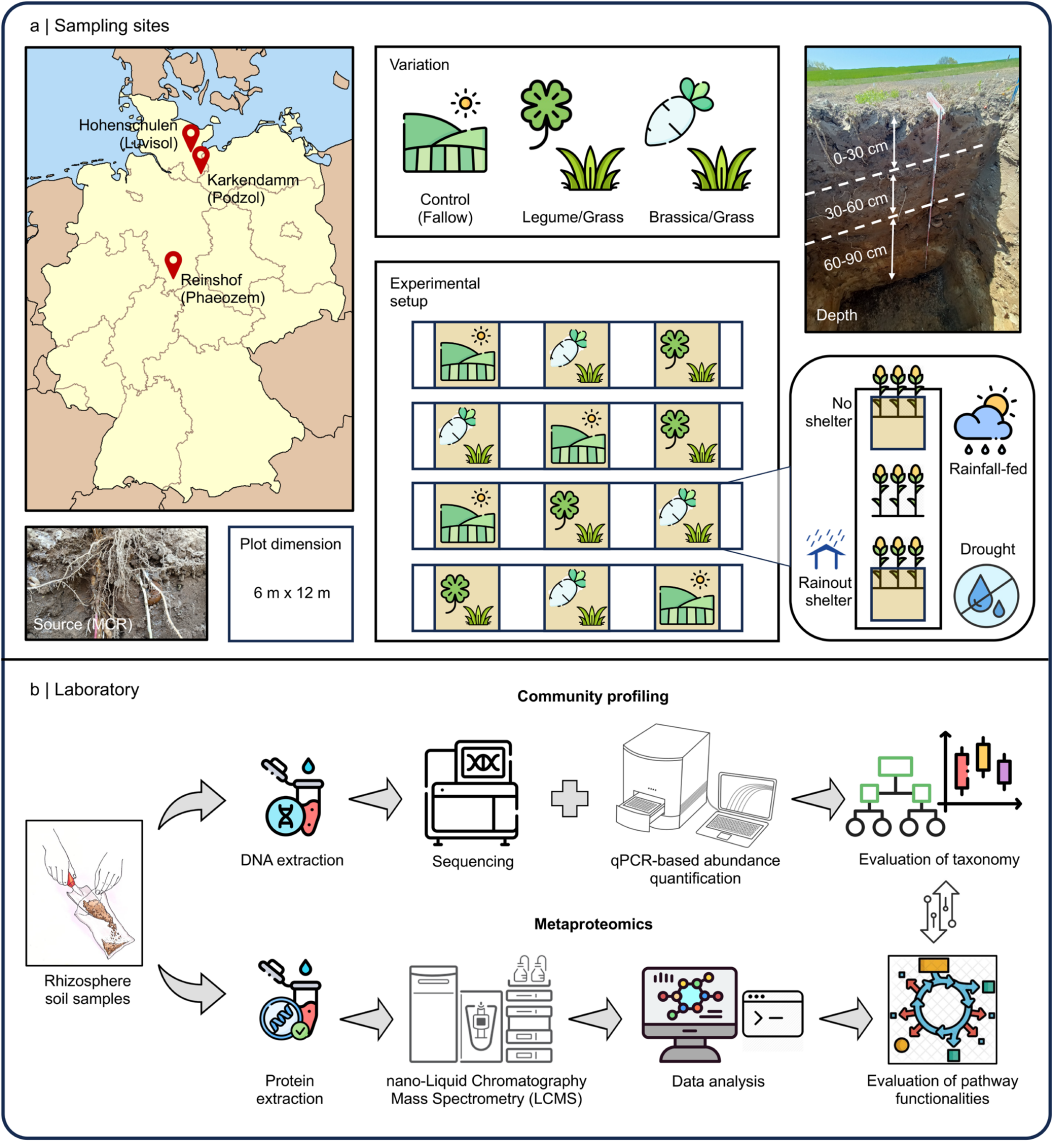
The experimental design for studying the reuse of winter cover crop root channels to cultivate maize. (a) Field sites: Samples from Luvisol, Podzol, and Phaeozem in Germany with a comparison of the bacterial microbiome associated with the roots of maize ( *Zea mays* L.) in root channels of cover crop [Legume/Grass (*Fabaceae*/*Poaceae*), Brassica/Grass (*Brassicaceae*/*Poaceae*)] or fallow (no cover crops) as control conditions. To replicate drought conditions, plots were covered with rainout shelters. (b) Community profiling using 16S rRNA gene‐based amplicon sequencing, qPCR, and metaproteomics was used to visualize how the composition and functional role of bacterial communities in the maize rhizosphere changed after being exposed to drought.

Crops were grown in three agricultural fields—at experimental estates Hohenschulen of the Christian‐Albrechts‐University of Kiel (Achterwehr, Germany, 54°18′44″N, 9°59′46″E), Karkendamm of the Christian‐Albrechts‐University of Kiel (Bad Bramstedt, Germany, 53°55′52″N, 9°55′15″E), and Reinshof of the Georg‐August‐University of Göttingen (Rosdorf, Germany, 51°29′05″N, 9°53′34″E). The cash crop in this study was maize ( *Zea mays* L.). Plots without cover crops during the winter (bare fallow) were established as controls and compared against two cover crop mixtures on distinct plots. The cover crops were shallow‐ and deep‐rooting *Brassicaceae* ( *Brassica napus* L., rapeseed, shallow‐rooting; *Raphanus sativus* L. var. *oleiformis*, oilseed radish, deep‐rooting); *Fabaceae* ( *Trifolium repens* L., white clover, shallow‐rooting; *Trifolium pratense* L., red clover, deep‐rooting); and *Poaceae* ( *Lolium perenne* , perennial ryegrass, shallow‐rooting; *Festuca arundinaceae*, tall fescue, deep‐rooting). The mixtures were grown as a combination of shallow‐ and deep‐rooting cover crops of *Brassicaceae*, *Fabaceae*, and *Poaceae*, complementing the niche complementarity principle, which has been reported to allow polycultures to overyield when plants compete for resources (Postma and Lynch 2012). All the cover crops were sown in October 2022 and grew until May 2023 in distinct randomized plots with four replicates of each variation. In May 2023, a herbicide formulation (Roundup, Bayer AG, Leverkusen, Germany) was applied to all experimental plots (including fallow plots) to kill all cover crops, and subsequently, maize was sown in the same plots with the cover crop variations in addition to the fallow plots. Maize was grown in the fields from May to August 2023 (Figure 1).

To compare drought‐like conditions against normal conditions, we artificially induced dry conditions using interrow rainout shelters and continued with the approach after observing differences in soil moisture trends between the sheltered and non‐sheltered profiles using time‐domain reflectometry sensors (Figure S1a–c). These specific types of rainout shelters covered half of the plot area between the maize rows, reducing 50% rainfall and restricting water infiltration to stemflow. The shelters were installed between the crop rows and below the maize canopy in June, at approximately 50 cm aboveground. This position ensured that the structures did not interfere with leaf‐level photosynthesis or direct light interception by the maize plants. They were constructed with a sloped configuration (higher at the front, lower at the back) to promote air flow and reduce the risk of heat accumulation or stagnant humidity. The plastic film used was standard greenhouse‐grade transparent polyethylene, which allows high light transmission while providing effective rain exclusion. Although we did not directly measure microclimatic variables such as air temperature under the shelters, the design was optimised based on preliminary tests to minimise shading and ventilation effects. A pictorial depiction of our rainout shelter setup used in the experiment has been provided as a Figure S2.

Prior to soil sampling, the soil profile was excavated to a depth of 1 m, then excavated 40 cm forward to obtain a fresh profile and fresh maize root system and to prevent any contamination by the neighboring soil. To compare the difference inflicted by drought on microbial communities in the reused root channels of cover crops by maize, we collected soil samples from a vertical soil profile from the topsoil (0–30 cm) and the subsoil (30–60 cm and 60–90 cm) for two different conditions: (1) maize roots growing in the cover crop root biopores (MCR) from those profiles subjected to drought (D), and (2) maize roots growing in the MCR in the profiles under rainfall‐fed conditions (RF). Maize rhizosphere soil collected along decayed cover crop roots was considered as originating from maize reusing cover crop root channels. The rhizosphere of maize and decayed cover crop roots was defined as extending 2 mm from the root surface. Therefore, the overlapping 2 mm rhizosphere zone of maize and decayed cover crop roots was designated as the sampling area. The sampling focused on clearly visible maize roots that were closely associated with remaining cover crop root residues, ensuring consistency in sample type across replicates and treatments to reduce subjective variation and avoid systematic error. Pictures of the reused root channels have been provided as a Figure S3. Additionally, maize rhizosphere samples from the control plot were collected, representing maize roots growing in bulk soil without reusing cover crop root channels. The sampling was done during the R1‐RX growth stage of maize (bolting) grown in the Luvisol (01.08.2023), the Phaeozem (19.07.2023), and the Podzol (26.07.2023), and the profiles were maintained until sampling was done around the flowering and reproduction stage of maize. The samples were extracted from the profiles using a spatula and collected in plastic zip‐lock bags. Until shipment to the laboratory, samples were stored in ice coolers containing dry ice in order to preserve bacterial communities and the metabolic picture for distinct sampling time points. In the laboratory, all samples were stored at −80°C until further processing. No repetition of sampling was done from the profiles of the same plot in order to avoid bias and duplicates. To minimize potential operator bias when identifying “root overlap regions”, we used a randomized sampling scheme with the four blocks representing the four replicates and the ambient (natural rainfall) and drought (with interrow rainout shelters) plots being always paired in direct proximity to each other.

### 2.2 Soil Physicochemical Properties

Soil microbial biomass carbon (C) and nitrogen (N) were determined using the chloroform fumigation extraction method (Sorkau et al. 2018; Vance et al. 1987). In brief, 7.5 g of soil was fumigated with chloroform for 24 h and then extracted with 30 mL of 0.05 M K_2_SO_4_ on a shaker for 1 h. C and N were measured with the N/C 2100 TOC/N analyser (Analytik Jena, Jena, Germany). MBC was calculated as the difference between extracted C from fumigated and non‐fumigated soil with a conversion factor (*k*_*C*_) of 0.45 (Joergensen 1996). MBN was calculated as the difference between extracted N from fumigated and non‐fumigated soil with a conversion factor (*k*_*N*_) of 0.54 (Brookes et al. 1985; Joergensen 1996). The MBC and MBN were presented as μg g^−1^ dry soil. Soil density and moisture content were also measured alongside.

Soil cylinder samples were taken to measure soil bulk density at each soil depth. Soil moisture content was also measured by water content sensors (Teros 10, Meter Group, München, Germany), which were installed in the control plots (in 0–30 cm, 30–60 cm, and 60–90 cm depth under rainout shelter and on the rainfall‐fed side) at each experimental site.

To investigate the influence of soil physicochemical properties on the bacterial microbiome in the maize root channels, we correlated the parameters pH, soil moisture content, TOC, TN, and soil bulk density with the 16S rRNA gene amplicon data using Partial Mantel's test (Mantel 1967) and confirmatory factor analysis (CFA) (Kline 2011) using the R package *microeco* (v1.10) (Liu, Cui, et al. 2020). An in‐depth explanation about the CFA and the equations implemented in this study is provided in the supplemental text. Additionally, the soil physicochemical properties were correlated to individual bacterial phyla along the different soil types and soil moisture conditions to understand the impacts of these factors on the individual communities.

### 2.3 DNA Extraction and Sequencing of 16S rRNA Gene Amplicons

Bacterial communities in the root‐vicinity samples were analyzed by sequencing of 16S rRNA gene amplicons (2 × 150 bp) on an Illumina NextSeq 550 (Illumina, San Diego, CA, USA). DNA was extracted from 0.25 g of soil using the DNeasy PowerSoil Pro Kit (QIAGEN GmbH, Hilden, Germany). PCR amplicons of the V3 region of the bacterial 16S rRNA gene were prepared with an established protocol (Vollmers et al. 2017) using the forward and reverse primers 341F and 518R and the NEBNext Ultra II Q5 Master Mix (New England Biolabs GmbH, Frankfurt, Germany). We chose the V3 region in order to avoid archaea and because the method was well‐established in our laboratories. Sequencing libraries were prepared from 100 ng of DNA according to the Illumina protocol. Dual index adapters for the sequencing were attached using the NEBNext Multiplex Oligos for Illumina. The final concentration of the libraries was 2 nM after pooling. We sequenced triplicates of samples from each soil depth and root‐vicinity source per cover crop variation plot for all three sampling sites (16S rRNA, *n* = 150).

The sequencing data were analyzed using QIIME2 v2023.5 (Bolyen et al. 2019). First, the raw sequence reads were demultiplexed and quality‐filtered (*q*‐score ≥ 25) using the q2‐demux plugin, followed by denoising with DADA2 (Callahan et al. 2016) (via q2‐dada2). Both the 16S forward and reverse sequences were trimmed at 150 bp. All amplicon sequence variants (ASVs) were aligned with mafft (Katoh et al. 2002) (via q2‐alignment), and then maximum‐likelihood trees were constructed using FastTree2 (Price et al. 2010) (via q2‐phylogeny). We chose ASV‐based methods over OTU approaches to allow for better comparability across studies and to limit the effect of spurious taxa on diversity indices (Reitmeier et al. 2021; Chiarello et al. 2022). Taxonomic assignment of bacterial ASVs was carried out using the q2‐feature‐classifier (Bokulich et al. 2018) and the classify‐sklearn Naïve Bayes taxonomy classifier against the Greengenes2 “99% OTU reference sequences” (McDonald et al. 2024). ASVs with a relative abundance of < 0.01% were defined as rare taxa. The identified bacterial phyla were categorized into r‐ and *K*‐strategists based on literature surveys (Davis et al. 2011; Fierer et al. 2007; Yin et al. 2022). Phyla comprising many r‐ and *K*‐strategists (e.g., *Pseudomonadota*) and unassigned ASVs were categorized as ‘r‐ or *K*‐strategist’. A list for the categorization of the bacterial communities into strategic groups has been provided as Table S1 with reasoning and references.

### 2.4 Quantitative PCR (qPCR)

The copy number of the 16S rRNA gene per gram of soil was quantified by SYBR Green‐based qPCR using a 7500 Fast Real‐Time PCR System (Applied Biosystems, Thermo Fisher Scientific, Waltham, MA, USA). Aliquots of the same DNA extract utilized in amplicon sequencing were used for qPCR. Dilutions of template DNA were used to compensate for the effect of PCR inhibitors in the samples. Each sample was analyzed in triplicate. PCR amplicon of the *Escherichia coli* V3 region was used as standards. Each 20 μL reaction contained 1 μL of template DNA, the forward and reverse primers 341F and 518R for 16S rRNA (Muyzer et al. 1993) without adapter nucleotides and Luna Universal qPCR Master Mix (NEB). Reaction conditions were an initial denaturation for 1 min at 95°C, followed by 40 cycles of denaturation at 95°C for 15 s and extension at 60°C for 30 s. The melting curve was recorded in the temperature range of 60°C to 95°C. The gene copy numbers per gram of soil were determined in comparison against the standard essentially as before (Adelowo et al. 2018). The average efficiency value was 97.2% ± 5.4%.

### 2.5 Metaproteomics Analysis

At each timepoint, samples were collected separately from three plots for each cover crop variation at the analysed soil depths and root‐vicinity sources and used for proteomic analyses following a previously described protocol (Ghosh et al. 2024) (*n* = 119). Approximately 4 g of soil (from the same set of samples used for 16S rRNA sequencing) was used for protein extraction using the SDS buffered‐phenol extraction method as previously described (Ghosh et al. 2024). The protein extract was purified using 1‐D SDS‐PAGE, and then the extract was further digested with trypsin. A nano‐HPLC system (UltiMate 3000 RSLCnano system, Thermo Fisher Scientific, Waltham, MA, USA) was used to separate the cleaved peptides. The system was connected to a Q‐Exactive HF Orbitrap LC–MS/MS system (Thermo Fisher Scientific) equipped with a nano electrospray ion source, Triversa NanoMate (Advion, Ithaca, NY, USA). We linked the MS data to an in‐house generated proteome database containing all the defined proteomes in UniProt for the bacteria identified in the samples by 16S rRNA amplicon sequencing. The database search was performed with Proteome Discoverer (v2.5.0.8, Thermo Fisher Scientific) using the SEQUEST‐HT algorithm, and all of the outputs are available on PRIDE (EMBL‐EBI) (Perez‐Riverol et al. 2025). The precursor mass tolerance of the MS was set to 10 ppm, and the fragment mass tolerance of the MS/MS was 0.02 Da. Carbamidomethylation of cysteine was considered fixed, and oxidation of methionine was set as a dynamic modification. Enzyme specificity was set to trypsin with up to two missed cleavages allowed using 10 ppm peptide ions and 0.02 Da MS/MS tolerances. Only rank‐one peptides with a Percolator‐estimated false discovery rate (FDR) < 1% were accepted as identified. The GhostKOALA and KEGG (Kanehisa et al. 2016) and COG (Galperin et al. 2015) databases were used for protein functional annotation. Pathways with a minimum of two proteins and a minimum coverage of 5% were selected for downstream processing. We analysed the measured label‐free quantification (LFQ) intensities of the identified proteins and determined the functional metabolic pathways to detect changes along the different sampling sites, soil moisture conditions, soil depths, and cover crop variations. For compiling the reference database, we selected protein sequences from the UniProtKB database based on the bacterial phyla identified in the 16S rRNA gene sequencing analysis. During the construction of databases from UniProt for mapping the identified proteins to their respective taxonomic communities, we made sure of minimum redundancy with maximum relevancy to negate repetitive identification of already measured proteins, which would skew the observations. In the analysis, proteins that were unambiguously identifiable by unique shared peptides and could be mapped back to the UniProt reference database were used for quantifications. Using the ENTREZ key, taxonomic identities were obtained from the NCBI database (Sayers et al. 2019; Schoch et al. 2020) for the identified individual proteins using KEGG's KO numbers as unique protein identifiers. Linking the datasets of identified proteins to the NCBI database provided us with all the taxonomic information regarding their sources. Any non‐bacterial proteins identified were filtered out and removed from our analysis. Each functional pathway had unique KEGG and COG identifiers, which were linked to proteins to connect the respective functional pathways and further use the NCBI‐linked KO identifiers for preparing integrated datasets of taxonomic communities and functional pathways. The LFQ values were highly variable for the identified protein groups, and so they were normalised by log2‐transformation using the *log* function of base R (v4.3.1) (R‐Core‐Team 2023) prior to any graphical representations or statistical significance tests. After the categorisation of proteins into different functional pathways, we tried to incorporate our observations into the specific cycles of interest in order to understand the changes inflicted by soil property and moisture content after the introduction of the re‐usage of cover crop root biopores and study the microbial response. Further, to observe if environmental stress impacted the biochemical pathways post‐root channel reuse, we quantified the fold change of LFQ intensities of identified proteins under drought against the proteins from rainfall‐fed conditions. The proteins were differentially regulated when the *Log2FC* was greater than 0.6 or less than −0.6 with significance *p* less than 0.05 (McCarthy and Smyth 2009; Peart et al. 2005; Raouf et al. 2008). Additionally, to evaluate the changes in the abundance of proteins of interest after introducing cover crop root channel re‐use, differential abundances of each protein were quantified, where their values for cover crop variations (*Brassicaceae*/*Poaceae* and *Fabaceae*/*Poaceae*) were subtracted from the values from fallow. Integrating the taxonomic information with protein datasets provided an overview of the role of these communities in the biochemical pathways with changeable soil composition and environmental conditions such as drought.

### 2.6 Statistical Data Analysis

We used R (v4.3.1) (R‐Core‐Team 2023) to perform all statistical analyses of the sequencing and the metaproteomics data. All measures of significance were calculated using multivariate analysis of variance (ANOVA), followed by Tukey's range post hoc test (TukeyHSD) with the package *stats* (v3.6.2) and *rstatix* (v0.7.2) (Kassambara 2023; Tukey 1953). In the 16S rRNA sequencing analysis, the ASV abundance tables were filtered with total‐frequency‐based filtering based on 95% sequence identity (via q2‐feature‐table summarize) and rarefied at 30,000 sequences to ensure equal sampling depth and sorting in the maximum number of samples for diversity analyses. Alpha and beta diversity metrics were calculated using the packages *phyloseq* (McMurdie and Holmes 2013), *metacoder*, and *microbiome* (Lahti and Shetty 2017) from R (v4.3.1) (R‐Core‐Team 2023). Shannon richness measured for each cover crop variation at different sampling sites and conditions was used for estimating alpha diversity richness. Pielou's evenness is the most widely used diversity evenness index in the ecological literature (Daly et al. 2018). For beta diversity, we used Jensen‐Shannon divergence (JSD) units (Lin 1991) and visualized differences via Principal Coordinate Analysis (PCoA) using the *vegan* package (v2.6–4) (Oksanen et al. 2022). Using a four‐way permutational multivariate analysis of variance (PERMANOVA), we evaluated the significantly different cover crop variations using the sampling sites, sampling conditions, and sampling depths as random effects and cover crop variations as the fixed effect. This was followed by Tukey's HSD for evaluating MANOVA test outcomes with parameters of cover crop variations, depth, sampling sites, soil moisture conditions, and bacterial phyla. For bacterial abundances under different parameters, the significance between the parameters was represented using the Compact Letter Display (CLD) (Piepho 2004) with the help of the *emmeans* package (Lenth 2025) since we can represent multiple pairwise significances using linear models. For metaproteomics, the significantly different cover crop variations or proteins of different metabolic pathways or bacterial phyla were calculated using MANOVA, using cover crop variations, sampling depths, soil moisture conditions, sampling sites, and bacterial phyla as fixed factors. Upon determination, they were represented by significant stars based on the adjusted *p*‐values (* *p* < 0.05, ** *p* < 0.01, *** *p* < 0.001, **** *p* < 0.0001). All figures were generated in RStudio using the packages *ggplot2* (v3.4.2) (Wickham 2016), *cowplot* (v1.1.1) (Wilke 2020), *circlize* (v0.4.16) (Gu et al. 2014), *WeightedTreemaps*, and *phyloseq* (v1.44.0) (McMurdie and Holmes 2013). Other integrated packages used for statistical analyses and figure generation were *tidyverse* (v2.0.0), *dplyr* (v1.1.3), and *splitstackshape* (v1.4.8) (Mahto 2013).

## 3 Results

### 3.1 Soil Types, Water Content, and Bacterial Community Diversity

The organic C content in the samples varied significantly with soil type and depth (Figure S4 and Table S2). Top‐ and subsoil total organic C (TOC) were highest in the Podzol, followed by the Luvisol and Phaeozem (topsoil: 1.76_Podzol_ > 1.27_Luvisol_ > 1.23_Phaeozem_; subsoil: 0.95_Podzol_ > 0.82_Luvisol_ > 0.59_Phaeozem_; units in g C kg^−1^soil). Total nitrogen (TN) content in the topsoil was the same across all three sites (0.13 g N kg^−1^ soil), but was higher in Luvisol subsoil than in the other two subsoil types (0.08_Podzol_ > 0.06_Luvisol_ ≥ 0.06_Phaeozem_; units in g N kg^−1^ soil). Soil pH varied considerably, with Podzol being the most acidic in topsoil and subsoil (both pH 5.21), followed by Luvisol (topsoil pH 6.49, subsoil pH 6.48), and Phaeozem being neutral to slightly basic (topsoil pH 7.14, subsoil pH 7.24). To assess the impact of water availability on the microbiomes, soil plots were subjected to RF or D conditions, achieved using rainout shelters. Gravimetric moisture content decreased under drought conditions for all soil types, but the reduction was most substantial in Podzol (5.58% RF to 3.85% D; 31‐fold% decrease), followed by Luvisol (16.16% RF to 14.34% D; 11.26‐fold% decrease) and the lowest in Phaeozem (30.42% RF to 29.46% D; 3.16‐fold% decrease).

**FIGURE 2:**
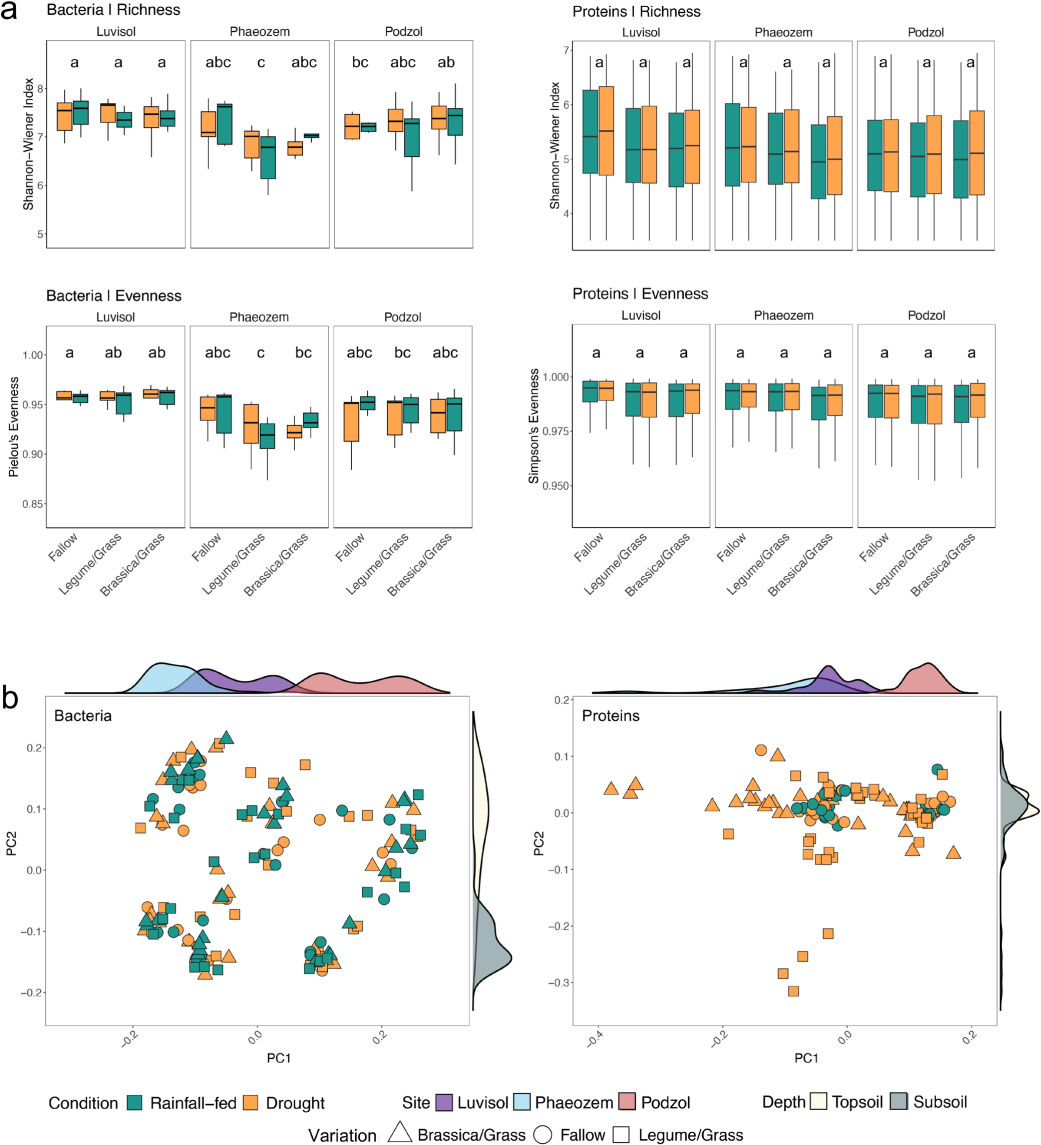
The taxonomic and functional diversity at the different experimental sites under soil moisture conditions. (a) Alpha diversity richness (Shannon–Wiener index) and evenness (Pielou's and Simpson's evenness index) metrics reflecting the distribution of bacterial communities and proteins at the three sampling sites under drought (D) and rainfall‐fed (RF) conditions. Pairwise correlation between the variations is shown using a compact letter display representation for the significant differences between the variations, calculated using Tukey's range test (TukeyHSD); (b) Bacterial community and proteomic beta‐diversity visualized using PCoA ordination based on Jensen‐Shannon divergence along the sampling sites and depths for each cover crop variation under soil moisture conditions. Distance metrics were calculated using amplicon sequence variants (ASVs) and unique clusters of orthologous genes (COGs). The different colors of the density plots next to the axes represent the soil types and sampling depths, and peak height and shape abundance. For Luvisol, *n* = 49; for Phaeozem, *n* = 50; and for Podzol, *n* = 51; for soil moisture conditions (drought and rainfall‐fed) and depth (topsoil and subsoil), *n* = 150 (statistical details in Table S3f–l).

We estimated the alpha‐ and beta‐diversity of the bacterial communities and detected proteins from the three soil types, sampling depths (topsoil and subsoil), and moisture conditions (D and RF) using 16S rRNA gene amplicon sequencing complemented by qPCR analysis and metaproteomics. Amplicon sequencing of 150 samples generated 42.8 million high‐quality reads, resulting in 117,483 unique amplicon sequence variants (ASVs) assigned to 132 classes within 42 bacterial phyla (Figure S5). Metaproteomics yielded a total of 2916 proteins belonging to 90 unique functional categories. Bacterial richness, as measured by the Shannon‐Wiener index, differed significantly with soil type (Figure 2a,b and Table S3a,b,i). Luvisol and Podzol exhibited higher richness (7.080_Podzol_ and 7.429_Luvisol_) than Phaeozem (6.956_Phaeozem_, *r* = 0.36, *** *p* < 0.001). Bacterial richness was also significantly greater in topsoil compared to subsoil (*r* = 0.36, *** *p* < 0.001), consistent with previous findings (Ghosh et al. 2024). In contrast, richness did not differ significantly between moisture conditions (7.151_D_ vs. 7.159_RF_, *r* = 0.36, *p* > 0.05), aligning with reports of limited drought impact on soil bacterial alpha‐diversity (Acosta‐Martínez et al. 2014; Tóth et al. 2017). Proteomic richness also varied significantly among soil types (4.735_Phaeozem_ vs. 4.500_Podzol_ and 4.839_Luvisol_, * *p* < 0.05, Figure 2a,b and Table S3c), but not between D and RF conditions (4.721_D_ vs. 4.661_RF_, *p* > 0.05, Figure 2a,b and Table S3h). Bacterial and proteomic evenness did not differ significantly across any of the studied parameters (*p* > 0.05, Figure 2a and Table S3d–e,h,j).

**FIGURE 3:**
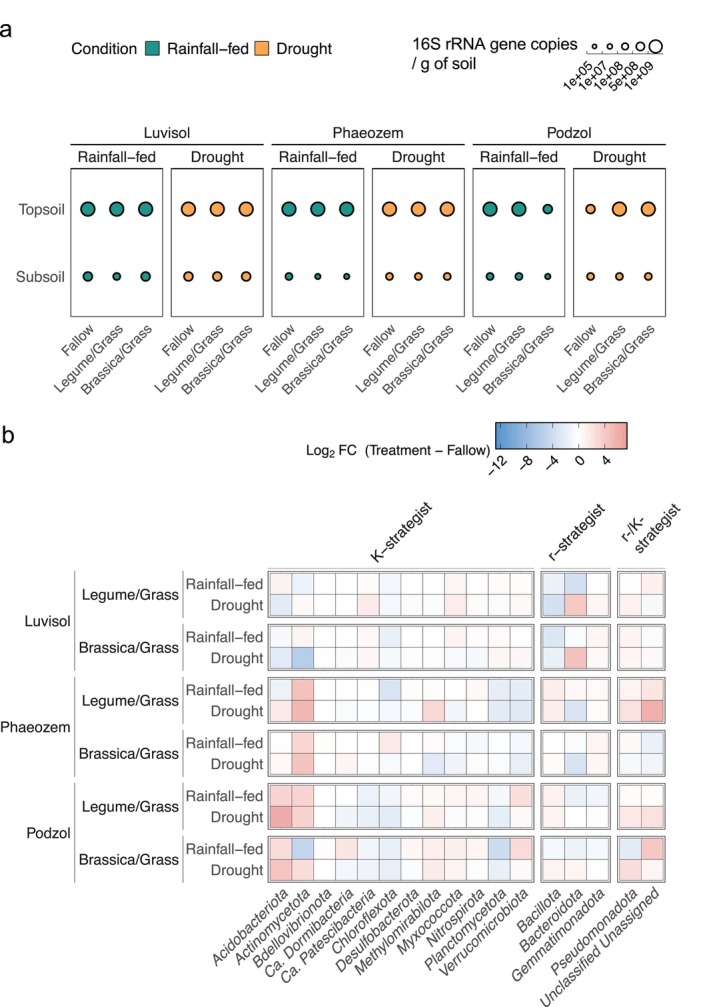
The absolute abundance of bacterial communities in the topsoil and subsoil at three soil types. (a) 16S rRNA gene copies measured using qPCR as a surrogate for absolute bacterial abundances in the soil types (Luvisol, *n* = 49; Phaeozem, *n* = 50; and Podzol, *n* = 51), of each cover crop variation (Fallow, Legume/Grass, and Brassica/Grass) and soil moisture conditions (drought (D) and rainfall‐fed (RF)). (b) Relative changes of each bacterial phylum (categorized as *K*‐strategist, r‐strategist, and r or *K*‐strategist) in reused cover crop root channels under D and RF conditions. Only phylotypes displaying a significant increase or decrease in abundance are shown.

Enumerations with qPCR revealed a decrease in the average number of 16S rRNA gene copies per gram of soil from 1.6 × 10^9^ ± 0.5 × 10^9^ in the topsoil to 3.1 × 10^8^ ± 2.3 × 10^8^ in the subsoil (Figure 3a; Table 1; Table S4a). In the topsoil, D conditions in the Podzol were associated with higher copy numbers in re‐used root channels from the *Brassicaceae/Poaceae* mixture compared to RF and decreased in the fallow setup under the same conditions. No significant differences in copy numbers were found between D and RF in root channels of the *Fabaceae/Poaceae* mixture or between topsoil of Luvisol and Phaeozem, regardless of cover crop mixture. In the subsoil, cover crop cultivation increased bacterial abundance in the Luvisol from the *Fabaceae/Poaceae* mixture and in the Phaeozem and Podzol from the *Brassicaceae/Poaceae* mixture under D vs. RF. As compared to fallow, abundances increased upon cover crop cultivation in the Luvisol and Phaeozem, while this effect was not observed in the Podzol. No differences in bacterial abundance were observed for subsoils between D and RF fallow treatments (Figure 3a; Table 1; Table S3h).

**TABLE 1:** Summary of 16S rRNA gene copies per gram soil for different parameters in our study. We quantified the mean and SD values of 16S rRNA gene copies per gram soil in soils from the reused cover crop root channels for all study parameters. The significance of each category is represented using compact letter display (CLD) calculated after conducting MANOVA on the gene copies under categories of soil type, cover crop variations and soil sampling depths. The values are provided in the Tables S3f–h and S4a.

Variation	Soil type	Depth	Condition	16S rRNA gene copies g soil^−1^ (mean + SD)	CLD
Fallow	Luvisol	Topsoil	Rainfall‐fed	1.77E + 09 ± 7.97E + 08	abcde
			Drought	1.48E + 09 ± 4.95E + 08	
		Subsoil	Rainfall‐fed	5.76E + 08 ± 9.99E + 08	def
			Drought	7.08E + 08 ± 4.20E + 08	
	Phaeozem	Topsoil	Rainfall‐fed	1.96E + 09 ± 2.06E + 08	ab
			Drought	1.88E + 09 ± 3.81E + 08	
		Subsoil	Rainfall‐fed	1.15E + 08 ± 6.06E + 07	f
			Drought	1.12E + 08 ± 7.43E + 07	
	Podzol	Topsoil	Rainfall‐fed	1.07E + 09 ± 5.27E + 08	cdef
			Drought	5.44E + 08 ± 3.01E + 08	
		Subsoil	Rainfall‐fed	1.11E + 08 ± 1.60E + 08	f
			Drought	4.45E + 08 ± 3.88E + 08	
Legume/Grass	Luvisol	Topsoil	Rainfall‐fed	1.41E + 09 ± 7.41E + 08	abcde
			Drought	1.93E + 09 ± 1.31E + 09	
		Subsoil	Rainfall‐fed	3.38E + 08 ± 4.31E + 08	f
			Drought	5.29E + 08 ± 1.10E + 09	
	Phaeozem	Topsoil	Rainfall‐fed	2.44E + 09 ± 1.06E + 09	a
			Drought	2.10E + 09 ± 4.85E + 08	
		Subsoil	Rainfall‐fed	8.97E + 07 ± 7.83E + 07	f
			Drought	2.08E + 08 ± 3.48E + 08	
	Podzol	Topsoil	Rainfall‐fed	1.74E + 09 ± 5.07E + 08	abc
			Drought	1.73E + 09 ± 4.24E + 08	
		Subsoil	Rainfall‐fed	1.10E + 08 ± 1.35E + 08	f
			Drought	1.92E + 08 ± 2.30E + 08	
Brassica/Grass	Luvisol	Topsoil	Rainfall‐fed	2.09E + 09 ± 1.08E + 09	abcd
			Drought	1.35E + 09 ± 6.61E + 07	
		Subsoil	Rainfall‐fed	6.86E + 08 ± 7.66E + 08	ef
			Drought	5.36E + 08 ± 5.21E + 08	
	Phaeozem	Topsoil	Rainfall‐fed	1.63E + 09 ± 6.58E + 08	abc
			Drought	1.87E + 09 ± 2.88E + 08	
		Subsoil	Rainfall‐fed	8.04E + 07 ± 6.38E + 07	f
			Drought	4.21E + 08 ± 4.09E + 08	
	Podzol	Topsoil	Rainfall‐fed	5.51E + 08 ± 2.64E + 08	bcdef
			Drought	1.53E + 09 ± 1.32E + 09	
		Subsoil	Rainfall‐fed	4.10E + 07 ± 4.94E + 07	f
			Drought	1.97E + 08 ± 2.79E + 08	

**FIGURE 4:**
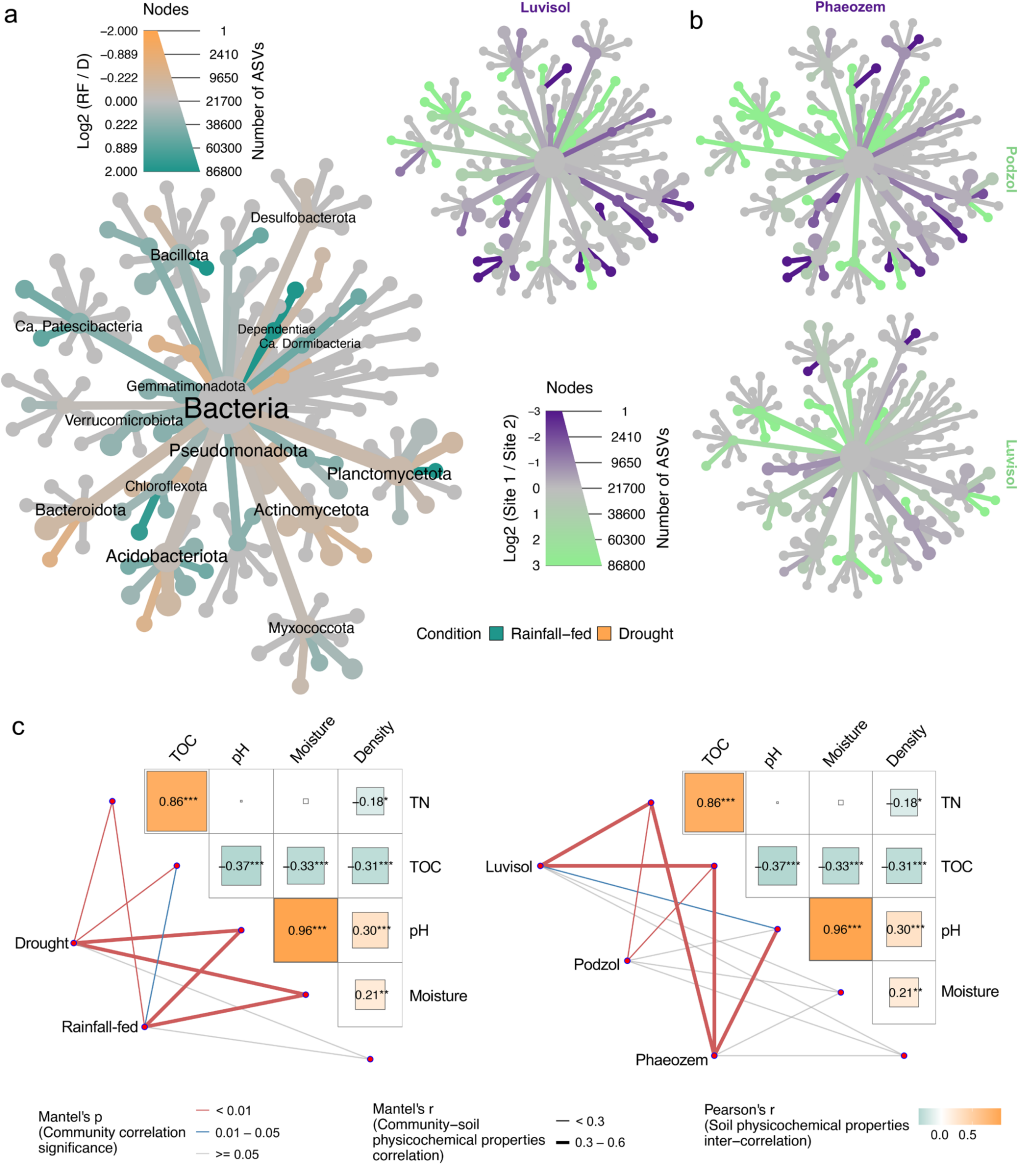
Correlations among soil physicochemical properties and bacterial community framework for soil types and moisture conditions. Taxonomic heat trees represent the differential abundance of the bacterial phylotypes between two parameters of (a) soil moisture content [drought (D) vs. rainfall‐fed (RF)], and (b) soil types (Luvisol, Phaeozem and Podzol). The yellow‐ochre colour indicates increases under D conditions and green colour under RF conditions, and dark green and purple have been used for pairwise comparisons among soil types. It is important to state that the annotation for (b) is the same as on (a) and so, no separate annotation has been represented for (b). (c) Partial Mantel's test: The matrix represents the correlation of the soil physicochemical parameters, the lines the correlation between the community distance matrix, and the thickness the strength of the correlation and the colour indicates the significance; * *p* < 0.05, ** *p* < 0.01, *** *p* < 0.001.

While alpha‐diversity did not differ between D and RF conditions, beta‐diversity varied significantly with soil types and sampling depths for both 16S rRNA gene amplicon and metaproteome data, as calculated using Jensen‐Shannon divergence coupled with PERMANOVA and Tukey's post hoc tests (Figure 2b and Table S3a). We describe the shifts in beta‐diversity using abundances of selected phyla (Tables S3f,g and S5). The changes were particularly pronounced for *K*‐strategists (Figures 3b and 4a; Table S4b). In cover crop root channels reused by maize roots, *Acidobacteriota* increased under D in the acidic Podzol but decreased under D in the Luvisol (*Log2FC*: 0.72_Podzol_ vs. 0.09_Phaeozem_ vs. −0.31_Luvisol_). *Actinomycetota*, another *K*‐strategist, increased in the neutral‐to‐basic Phaeozem under D (1.90_Podzol_ vs. 1.38_Phaeozem_ vs. 0.58_Luvisol_). *Planctomycetota* increased in the Podzol (0.97_Podzol_ vs. 0.06_Phaeozem_ vs. 0.07_Luvisol_) under D, whereas *Pseudomonadota* increased in all three soil types under D (1.01_Podzol_ vs. 1.08_Phaeozem_ vs. 0.14_Luvisol_), which is similar to previous findings under dry conditions (Ngumbi and Kloepper 2016). In contrast, *Chloroflexota* and *Verrucomicrobiota* decreased under D (−0.23_*Chloroflexota*_ and −0.41_*Verrucomicrobiota*_) compared to RF conditions. Changes in the abundance of 11 out of the 132 identified bacterial classes differed from that of their respective phylum (e.g., *Symbiobacteria* of *Bacillota* and UBA8108 of *Planctomycetota*) during drought, exemplifying the notion that members of a bacterial phylum do not always share similar ecophysiological traits (Morrissey et al. 2016). *Ca*. Dormibacteria, a newly characterised taxon that is also considered a class of *Chloroflexota* and known to favour dry, nutrient‐poor soils (Montgomery et al. 2021), was abundant in sandy Podzol and in the deeper subsoil (12‐fold increase in abundance in Podzol compared to Luvisol and Phaeozem; 7.4‐fold increase in subsoil as compared to topsoil, Figure 4b and Table S4b).

### 3.2 Comparing Soil Physicochemical Properties

**FIGURE 5:**
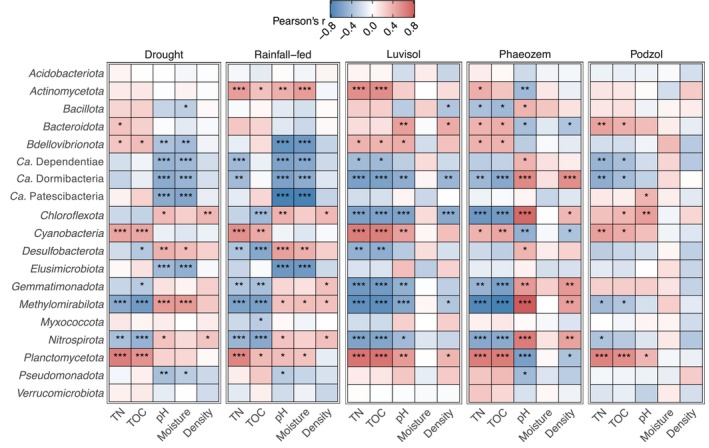
Correlation of individual bacterial communities and the soil physicochemical properties. The correlations between each bacterial phylum and the physicochemical properties (TN, TOC, pH, Moisture, and Density) have been estimated using Partial Mantel's test. Pearson's correlation values and the significance estimation for each physicochemical property and each phylum are provided in the Table S6e,f. The significant correlations are represented by asterisks inside the heatmap tiles; * *p* < 0.05, ** *p* < 0.01, *** *p* < 0.001.

Partial Mantel tests revealed significant relationships between community structure and pH, soil moisture content, C and N (** *p* < 0.01, Figures 4c and 5; Table S6a). The strength of the correlation between community composition, moisture, and pH decreased under D as compared to RF (r_pH,D_ = 0.473 vs. r_pH,D_ = 0.525, r_H2O,D_ = 0.416 vs. r_H2O,RF_ = 0.483, Table S6b).

CFA modelling further supported that pH (0.71_CFA_), soil moisture content (0.07_CFA_), and bulk density (0.10_CFA_) were key physicochemical factors influencing community composition, with a strong connectedness between the three factors (Figure S6, *** *p* < 0.001, Tables S6c and S7). The modelling also indicated that cover crop roots provide increased access to total soil organic carbon (Figures S7 and S8; Table S6c) (Aguilera et al. 2013; Hu et al. 2024). The effect of blocking precipitation via rainout shelters on the bacterial communities diminished with depth (r_Topsoil_ = 0.704, r_Subsoil_ = 0.581, Figures S9 and S10; Table S6d), potentially due to reduced drought stress and more stable water content in the subsoil. At the community level, *Cyanobacteria* and *Planctomycetota* abundances positively correlated with TOC and TN (Figure 5 and Table S6e), consistent with previous reports (Liu et al. 2016; Qu et al. 2018). Conversely, *Chloroflexota*, *Methylomirabilota*, and *Nitrospirota* exhibited negative correlations with TOC and TN across all three soil types (Figure 5 and Table S6f).

Lower TOC and TN in the subsoil appeared to favor *Chloroflexota*, *Methylomirabilota*, and *Nitrospirota*, potentially allowing them to outcompete other phyla (Figure 5; Figures S5 and S9; Tables S2, S6e,f, S7). Similarly, members of rare taxa such as *Ca*. Dependentiae and *Ca*. Patescibacteria (also known as Candidate Phyla Radiation) increased relatively in the subsoil compared to the topsoil in reused cover crop root channels (Figure 5; Figures S5 and S9; Tables S2, S6e,f, S7). Additionally, the correlation between *Methylomirabilota* and *Ca*. Patescibacteria with moisture was stronger under D than under RF conditions (Figure 5).

### 3.3 Functional Response of Root Channel‐Residing Bacteria Towards Drought

Metaproteomic analysis revealed the functional response of bacterial communities within maize root channels to D conditions. Protein group abundances varied significantly with soil type, depth, and cover crop variation and were consistent with 16S rRNA gene amplicon sequencing results (Table S8a). Samples from the Podzol consistently exhibited the highest abundance, followed by Phaeozem and Luvisol (Table S8b). More protein groups were found in topsoil compared to subsoil across all soil types (582_Podzol‐Topsoil_ > 455_Phaeozem‐Topsoil_ > 259_Luvisol‐Topsoil_; 330_Podzol‐Subsoil_ > 262_Phaeozem‐Subsoil_ > 188_Luvisol‐Subsoil_) and in D compared to RF samples (443_D,avg_. > 355_RF,avg_.). Protein expressions in D vs. RF increased in the Podzol and the Phaeozem in reused cover crop root channels (0.677_Podzol‐*Log2FC*_, 0.576_Phaeozem‐*Log2FC*_), but decreased in the Luvisol (0.665_Luvisol‐*Log2FC*_), and did not significantly change in fallow, assessed by Wilcoxon and multivariate analysis of variance (MANOVA) tests (Table S8c,d). Root channels from the *Brassicaceae*/*Poaceae* mixture exhibited the greatest variability compared to *Fabaceae*/*Poaceae* and fallow, possibly reflecting differences in soil physicochemical properties of their pore walls, such as water content, bulk density, and C and N availability. Detailed metadata of all identified proteins and the associated biochemical pathways are provided in Table S9.

**FIGURE 6:**
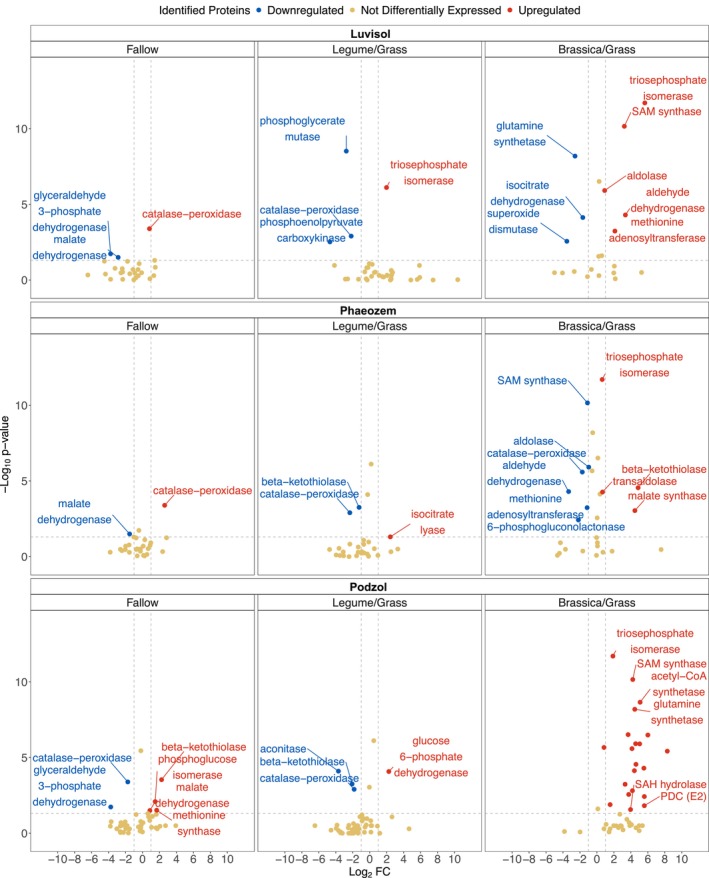
Upregulated and downregulated proteins under drought. A volcano plot representation for the upregulation and downregulation of the identified proteins under drought conditions at the three sampling sites (Luvisol, Phaeozem, and Podzol) for the cover crop variations (Fallow, Legume/Grass, and Brassica/Grass). The *x*‐axis represents the Log2 fold‐change, and the *y*‐axis represents the −Log_10_ *p*‐value according to the Mann–Whitney *U* test. The proteins having Log2 fold‐change (FC) values < 0.6 or > −0.6 and *p* > 0.05 were classified as ‘Not Differentially Expressed’. Log2FC > 0.6 and *p* < 0.05 were the ‘Upregulated’ proteins, and Log2FC< 0.6 and *p* < 0.05 were the proteins ‘Downregulated’. Names of several identified proteins are not shown to avoid crowdedness. The information for all proteins is provided in the Table S10.

**FIGURE 7:**
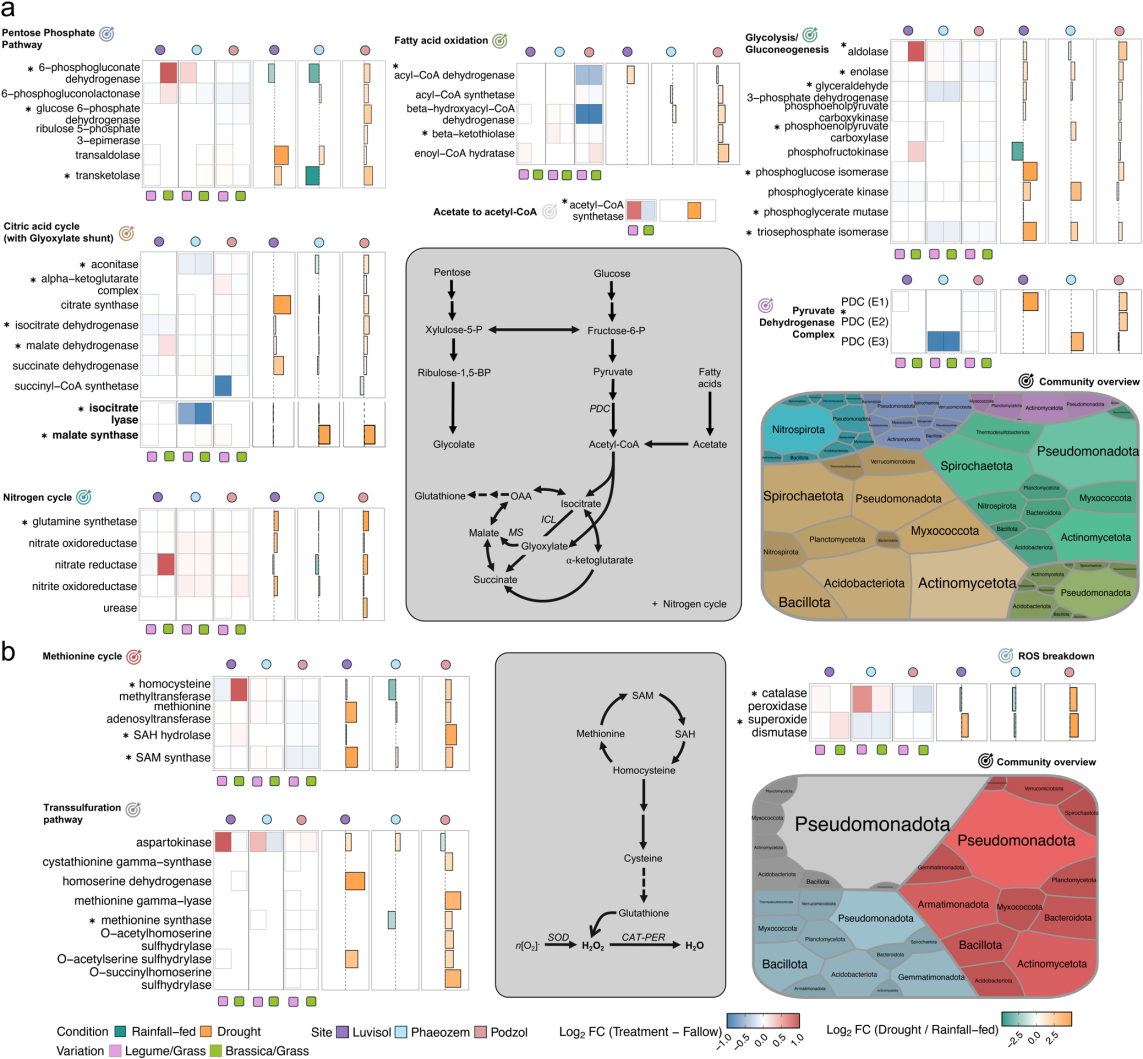
Overview of the functional pathways affected by drought in three soils after cover crop root‐channel‐reuse. Pathways with contributions to (a) catabolism to (b) anabolism. Heatmaps are showing the net expression change based on measured protein LFQ intensities for each protein identified. Positive logarithmic values of the difference are denoted by red (higher in re‐used root channel than in bulk soil rhizosphere) and negative values by blue (lower in re‐used root channel than in bulk soil rhizosphere). The bar charts represent whether the proteins were differentially expressed under drought. The small circles and boxes at the top and below the heatmaps and bar charts code for the soil types and cover crop mixtures respectively. Tree maps represent the bacterial phyla identified as pathway source. The significance of the changes in protein expression under drought conditions at each site was calculated using a four‐way ANOVA model, significant changes in expression are marked with an asterisk (* *p* < 0.05) (Table S11a consists of a list of proteins which represented significant changes across the parameters of study).

Volcano plot analysis identified differentially expressed enzymes under D stress, with a greater number of upregulated proteins in the root channels of *Brassicaceae/Poaceae*, especially in the drought‐affected Luvisol and Podzol soils (Figure 6 and Table S10). The upregulated enzymes were primarily involved in glucose and pyruvate metabolism, the glyoxylate shunt of the citric acid cycle (TCA), amino acid synthesis, the methionine cycle, and the transsulfuration pathway (Figure 7a; Tables S10 and S11a). Specifically, several glycolytic enzymes, including aldolase, enolase, phosphoglucose isomerase, and triose phosphate isomerase, were significantly upregulated under D in the Luvisol and Podzol (* *p* < 0.05, Tables S10 and S11b). Upregulation of phosphoenolpyruvate carboxykinase (*** *p* < 0.001, Tables S10 and S11a) likely facilitated the use of oxaloacetate for pyruvate synthesis and subsequent gluconeogenesis. Expression of enzymes from the pentose phosphate pathway, 6‐phosphogluconate dehydrogenase (6PGDH) and glucose‐6‐phosphate dehydrogenase (G6PDH), increased under D (*** *p* < 0.0002), matching previous studies with rice, tomato, and soybean roots but without cover cropping (Hou et al. 2007; Landi et al. 2016; Wang et al. 2020). 6PGDH and G6PDH were upregulated in the Podzol and in the reused root channels of *Brassicaceae/Poaceae* in the Luvisol, whereas 6PGDH was downregulated in the Phaeozem (Figure 7a). Elevated expression of pyruvate dehydrogenase complex proteins and acetyl‐CoA synthetase under D conditions in all the soil types suggests increased synthesis of acetyl‐CoA from pyruvate, acetate, and fatty acid oxidation, potentially promoting the glyoxylate shunt, a TCA cycle variant favored under such conditions (Ahn et al. 2016) (Figure 7a). Upregulation of the glyoxylate shunt was evidenced by significantly increased abundance of malate synthase, which converts glyoxylate to malate. Catalase‐peroxidase (CAT‐PER) and superoxide dismutase (SOD) abundances were higher in the Luvisol and the Podzol compared to the Phaeozem, suggesting enhanced reactive oxygen species (ROS) scavenging (*** *p* < 0.001, Figure 7b). In the Podzol, the majority of enzymes involved in the methionine cycle and transsulfuration pathway were also overexpressed (** *p* < 0.01). These two pathways produce glutathione, needed for facilitating CAT‐PER‐based breakdown of ROS (Figure 7b; Tables S10 and S11a). Enzymes contributing to amino acid synthesis, including 1‐pyrolline‐5‐carboxylate dehydrogenase, aldehyde dehydrogenase, branched‐chain transaminase, and glutamate‐gamma semialdehyde dehydrogenase, were also more abundant under D conditions across all three soil types (Figure S11). In the N cycle, glutamine synthetase was the only enzyme that showed a differential expression, specifically an upregulation in the Podzol and downregulation in the Luvisol and Phaeozem under D compared to RF conditions with *Brassicaceae/Poaceae* as the winter cover crops (Table S10).

Comparing relative protein expression between cover crop mixtures and fallow treatment revealed greater variability and upregulation in topsoil under D versus RF conditions, while subsoil expressions were similar under both conditions (Figure S12; Tables S8e and S10). Protein expression was significantly higher in topsoil compared to subsoil (2.11‐fold increase, Table S8e). The identified bacterial proteins were predominantly from *K*‐strategists. In topsoil, the phyla *Acidobacteriota*, *Actinomycetota*, *Armatimonadota*, *Myxococcota*, *Planctomycetota*, *Pseudomonadota*, and *Verrucomicrobiota* contributed the highest abundances of identified proteins (Figures S13–S15, Table S9). In contrast, *Bacillota*, *Chloroflexota*, *Methylomirabilota*, and *Thermodesulfobacteriota* were the dominant sources of identified proteins in the subsoil (Figures S13–S15 and Table S9). Enzymes of the glyoxylate shunt, cysteine and methionine metabolism, and ROS breakdown were mostly from *Methylomirabilota*, *Pseudomonadota*, *Spirochaetota*, and *Verrucomicrobiota* (Figures S13–S15 and Table S9).

**FIGURE 8:**
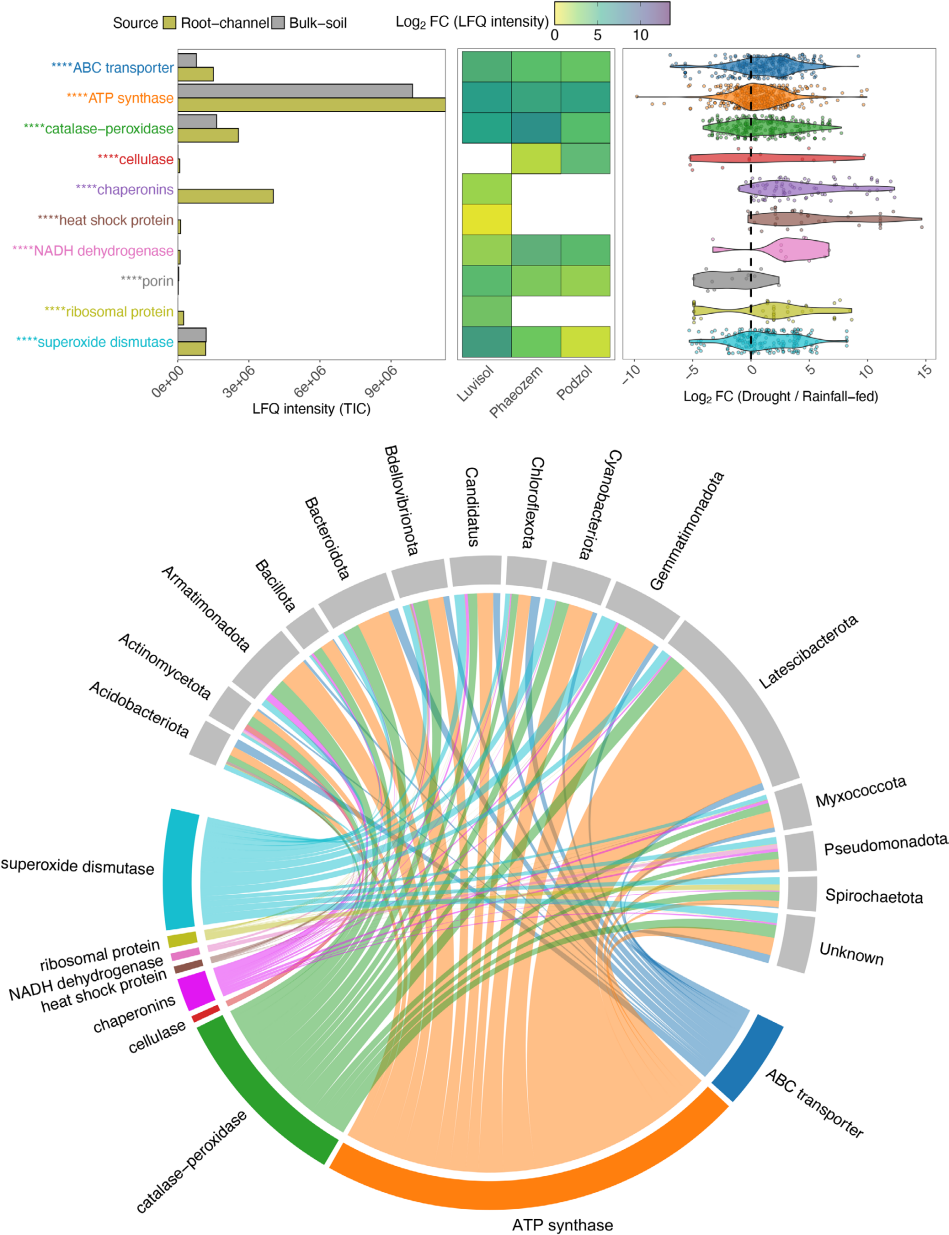
Estimation of bacterial activity. Chord diagram showing abundance changes of predominant proteins (chaperonins, ribosomal proteins, ROS regulators, membrane proteins such as porins, ATP synthase). Substantial abundance changes indicate high translation activity and hence overall metabolic activity. Protein presence in the three different soil types is represented by the heatmap, followed by the comparative abundance under drought conditions as compared to rainfall‐fed conditions, represented as a violin plot. Proteins showing significant changes in expression are marked with an asterisk (**** *p* < 0.0001).

The expression of proteins indicative of general metabolic activity, including ribosomal proteins, chaperonins, transmembrane proteins, and peroxidases, was significantly higher in reused cover crop root channels compared to fallow (*** *p* < 0.001) (Figure 8 and Table S12a,b). Under D conditions, the abundance of these proteins increased by multiple orders in root channels as compared to RF (21.9‐fold increase in chaperonins; 16.9‐fold increase in heat shock proteins and 17.9‐fold increase in ribosomal proteins), with ATP synthase being the most abundant protein detected, which increased by 0.2‐fold under D against RF. The majority of these proteins were sourced from the rare phyla *Armatimonadota* and *Ca*. Latescibacterota.

## 4 Discussion

This study investigated the impact of drought on bacterial communities and expressed biochemical pathways in winter cover crop root channels reused by maize. The reuse of cover crop root channels facilitates maize root propagation into the subsoil, likely by reducing penetration resistance and potentially by increasing nutrient availability in the channel wall accessible to the bacterial community beneficial to maize (Ghosh et al. 2024). Here, increased nutrient availability can support the greater copy numbers of the 16S rRNA gene in reused cover crop root channels, particularly from *Brassicaceae/Poaceae* in Podzol, compared to maize root channels in fallow. Building upon previous research examining drought effects on soil biogeochemistry and microbial communities (Bogati and Walczak 2022; Deng et al. 2021), our findings reveal relationships between the type of cover cropping, soil type, moisture availability, and bacterial microbiome dynamics and function.

Bacterial alpha‐diversity and protein distributions differed significantly among soil types (Luvisol > Podzol > Phaeozem) but were not affected by soil moisture conditions. The observed higher richness in drought‐impacted Luvisol and Podzol soils may reflect the legacy of decade‐long water scarcity in the region (Boeing et al. 2022), consistent with recent reports of increased bacterial richness under prolonged drought (de Souza et al. 2024). We observed a greater prevalence of *K*‐strategists (e.g., *Acidobacteriota*, *Actinomycetota*, and *Planctomycetota*) across our samples, consistent with the resilience of slow‐growing bacteria to environmental stress (Li et al. 2021; Liu et al. 2024; Naylor and Coleman‐Derr 2018). These bacteria are better adapted to resource‐limited conditions and can maintain activity under adverse conditions, possibly outcompeting the r‐strategists, which require resource‐rich conditions and are not able to cope with D stress. Conversely, the decline in oligotrophic phyla (*Chloroflexota*, *Ca. Dependentiae*, *Methylomirabilota*, *Ca. Patescibacteria*) in the topsoil can be potentially due to increased O_2_ levels in root channels under D conditions (Figures 3b and 4a; Figure S9; Table S4c,d). These phyla generally thrive in oxygen‐deficient environments such as water‐filled pore space (Aguado‐Norese et al. 2023; Dai et al. 2019; Ghosh et al. 2024), and the drying of the topsoil, especially of the Luvisol and the Podzol, results in increased aeration. In spite of the increased prevalence of *K*‐strategists, similar bacterial evenness at the three sites under D conditions is a plausible representation of altered community composition, where drought‐tolerant taxa replace the intolerant ones, and if the communities are functionally redundant, that would explain the unchanged proteomic richness and evenness (Ochoa‐Hueso et al. 2018). The incorporation of cover crops might be helping in maintaining the bacterial framework since previous research has observed drought stress to significantly impact soil microbial activity (Bogati and Walczak 2022). The increased abundance of *Ca. Dependentiae* and *Ca. Patescibacteria* in the subsoil following cover crop cultivation, potentially due to exploiting stimulated rhizodepositions (Chaudhari et al. 2021) (Table S4d). This is likely facilitated by the ability of these bacteria to prosper under low‐nutrient, low‐moisture conditions, aligning with reported inverse relationships between their abundance and soil moisture (Naylor and Coleman‐Derr 2018; Yang et al. 2024). This also explains their prominence in the Podzol, which has significant aeration because of the significant sand composition. The acidic‐to‐slightly acidic conditions of the Podzol and the Luvisol favored the proliferation of *Acidobacteriota*, *Actinomycetota*, *Armatimonadota*, and *Bacteroidota*. The latter two phyla exhibit tolerance to lower soil moisture, potentially contributing to their increased abundance under D conditions (Kruczyńska et al. 2023; Sait et al. 2006). Furthermore, we observed a drought‐induced shift towards phyla adapted to nutrient‐poor conditions in the Luvisol and Podzol, including *Ca. Dormibacteria*, *Methylomirabilota*, and *Nitrospirota*, at the expense of *Chloroflexota*. While the drier channels during drought may have greater availability of O_2_, reduced water content, and limited nutrient diffusion, favoring phyla better adapted to nutrient scarcity. Phaeozems, characterized by high soil organic matter content, supported the proliferation of organic matter decomposers such as *Actinomycetota* (Silva et al. 2022), which thrive across a broad pH range (Shivlata and Tulasi 2015). Conversely, conditions in Phaeozems may lead to *Planctomycetota*, which are adapted to nutrient‐deficient environments (Kaboré et al. 2020), being outcompeted by other bacteria.

Physicochemical properties in the rhizosphere of the reused root channels, including pH, moisture content, total nitrogen, and organic carbon, are key determinants of soil quality and bacterial community structure. While previous studies have demonstrated variable relationships between these properties, we observed a positive correlation between pH and soil moisture, potentially linked to changes in C metabolism and proton release during nitrification as soils dry (Slessarev et al. 2016; Zárate‐Valdez et al. 2006). The mobilization and transport of organic matter from one part of the soil profile to another is only known for the Podzol due to the acidic nature of the soil. The drying up of water from the soil pores hinders organic matter mobility and increases their accumulation, which may explain the negative association between pH and organic carbon (Orme et al. 2022; Sauer et al. 2007). Drought‐induced increases in soil aeration also expose previously inaccessible carbon sources to aerobic bacteria, probably initiating its mineralization (Deng et al. 2021; Vahedifard et al. 2024).

To investigate these relationships further, we measured the abundance of proteins across root channels from the Luvisol, Podzol, and Phaeozem soils under D and RF conditions using metaproteomics. Our analysis revealed distinct responses to drought, with upregulation of glycolytic and pentose phosphate pathway enzymes observed in the drought‐affected Luvisol and Podzol, but not in the Phaeozem, which maintained higher moisture levels. It has been previously documented that both the Luvisol and the Podzol have a high composition of sand, whereas the Phaeozem features a substantial silt composition (Grosse et al. 2021; Voelkner et al. 2019), complementing the observations. The observed increased expression of glycolytic enzymes in the Luvisol and the Podzol reflects a plausible increase in substrate availability due to enhanced aeration in the drier soil leading to the utilization of previously sequestered TOC and a potential contribution to elevated TOC contents (Table S2) (Melillo et al. 2002; Zhang et al. 2014). Wang et al. previously reported increases in soil microbial C and N biomass under short‐term drought due to litter decomposition by soil microorganisms (Wang et al. 2023). In our case, this could increase the availability of substrates—ultimately leading to upregulated glycolytic enzymes and the prevalence of respective enzyme‐producing bacteria. The increased bacterial activity was further manifested by elevated levels of the pyruvate dehydrogenase complex, acetyl‐CoA synthetase, and enzymes for β‐oxidation of fatty acids. Consistent with these findings, ^13^C labelling studies have reported an accumulation of two‐carbon compounds, such as acetate, in drought‐affected soils (Kornberg and Krebs 1957; Werner et al. 2020). Increased availability of acetate and metabolism of fatty acids would also explain the higher abundance of enzymes of the glyoxylate cycle, malate synthase and isocitrate lyase in the Podzol (Koedooder et al. 2018). Furthermore, there was an increased abundance of carbohydrate‐active enzymes (CAZymes), particularly β‐glucosidase, from *Bacteroidota* in *Brassicaceae/Poaceae* root channels of the Luvisol (Figure S16).

The pentose phosphate pathway also contributes to NADPH generation needed for maintaining cellular redox homeostasis mechanisms, specifically in the G6PDH and 6PGDH reactions (Fuentes‐Lemus et al. 2023). Both enzymes were upregulated under D conditions in the Podzol and in the reused root channels of *Brassicaceae/Poaceae in the Luvisol*, the two soil types experiencing higher D stress leading to a potentially higher NADPH generation, which was not the case for 6PGDH in Phaeozem. The NADPH is utilized by enzymes such as CAT, PER, glutathione peroxidase (GPX), and SOD to mitigate ROS‐induced stress, which likely occurs during drought within the well‐aerated root channels. Methionine cycle enzymes *S*‐adenosylmethionine synthase and *S*‐adenosylhomocysteine hydrolase catalyze *S*‐adenosylmethionine to generate polyamines, which have been associated with abiotic stress regulations, previously reported in plants (Gong et al. 2014). Similarly, upregulation of transsulfuration pathway enzymes leads to generating more cysteine towards glutathione synthesis (Xi et al. 2025), which subsequently acts as cofactors to the reduction of H_2_O_2_ into H_2_O by GPX. This explains the upregulation of enzymes of both these pathways in the drought‐prone Podzol. Furthermore, the increased abundance of aldehyde dehydrogenase, branched‐chain transaminase, and glutamate‐gamma‐semialdehyde dehydrogenase in the Luvisol and Podzol suggests enhanced amino acid synthesis, particularly glutamate and proline, which function as osmoprotectants for bacteria living in shrinking water films during drought (Figure S11). The expression of these pathways was particularly pronounced in the topsoil, likely due to its direct exposure to drought stress, while the subsoil exhibited greater stability in protein expression. The observed upregulation of enzymes involved in the glyoxylate cycle and ROS scavenging in all the soil types suggests that aerobic bacteria, particularly members of the *Pseudomonadota*, *Spirochaetota*, and *Verrucomicrobiota* phyla, respond to these stresses more strongly than other phyla. These enzymes can, therefore, represent plausible indicators of drought stress in affected soil profiles.

The increased abundance of proteins associated with membrane function, ribosomes, and electron transport following maize root colonisation of former cover crop channels suggests heightened bacterial activity. This observation supports the previous finding that root channels created by cover crop roots facilitate the formation of microbial hotspots within the maize rhizosphere (Kuzyakov and Blagodatskaya 2015). Notably, the significant contribution of rare taxa—bacterial phylotypes with a relative abundance below 0.01%, including members of *Armatimonadota* and Ca. Latescibacterota—indicates increased and diversified microbial activity. This diversification likely stems from the complex composition of organic matter within these channels, a mixture of decaying root detritus and microbial necromass from the previous cover crop rhizosphere, combined with fresh root exudates from maize. This complex substrate base likely enhances access to subsoil resources (Ghosh et al. 2024). The cover crop mixture with *Brassicaceae* and *Poaceae* exhibited the most pronounced upregulation of these pathways, particularly in rapidly draining soil types like the Luvisol and the Podzol. *Brassicaceae* are known for establishing deep root channels and efficiently exploiting subsoil resources, even during short‐term winter cover cropping. Consequently, the microbiome inhabiting these channels may be pre‐adapted to utilize subsoil resources, potentially buffering maize against drought‐induced stresses.

Understanding the full complexity of microbial interactions within root channels remains a significant challenge. The walls of reused cover crop root channels represent an open system characterized by diverse microhabitats and influenced by fluctuating physicochemical factors like the differences observed for the soil organic carbon content. These factors also contribute to the complexity of linking soil compositions with the microhabitat inhabitants. We observed that certain bacterial phylotypes, such as *Symbiobacteria* (*Bacillota*) and UBA8108 (*Planctomycetota*), exhibited abundances inconsistent with broader patterns of their respective phyla, illustrating the nuanced dynamics and potentially different ecophysiological traits of these microorganisms. Integrating community profiling with metaproteomics, coupled with the continued expansion of reference databases, will be crucial for resolving these complexities. Overall, our approach successfully generated a substantial proteomic dataset, providing a functional overview of biochemical pathway shifts under D conditions and revealing the key roles played by specific bacterial community members. Our findings demonstrate that drought stress elicits a coordinated bacterial response in the rhizosphere of maize reusing cover crop root channels, characterized by upregulation of the glyoxylate cycle and enzymes involved in ROS scavenging. The observed responses are particularly pronounced in the topsoil and modulated following *Brassicaceae* and *Poaceae* cover crops. These results highlight the potential for leveraging cover cropping strategies to mitigate the impacts of drought on agricultural systems.

## Author Contributions

**Debjyoti Ghosh:** data curation, formal analysis, investigation, methodology, software, validation, visualization, writing – original draft, writing – review and editing. **Yijie Shi:** data curation, investigation, writing – review and editing. **Iris M. Zimmermann:** methodology, project administration, resources, writing – review and editing. **Katja Holzhauser:** data curation, investigation. **Martin von Bergen:** resources. **Anne‐Kristin Kaster:** resources. **Sandra Spielvogel:** conceptualization, methodology. **Michaela A. Dippold:** conceptualization, methodology, writing – review and editing. **Jochen A. Müller:** conceptualization, methodology, resources, supervision, writing – review and editing. **Nico Jehmlich:** conceptualization, funding acquisition, methodology, resources, supervision, writing – review and editing.

## Conflicts of Interest

The authors declare no conflicts of interest.

## Supporting information


**Figures S1‐S16:** gcb70512‐sup‐0001‐Text‐FiguresS1‐S16.zip.

**Tables S1‐S13:** gcb70512‐sup‐0002‐TableS1‐S13.xlsx.

## Data Availability

The raw sequencing data and the respective metadata generated from this study are available under the NCBI BioProject ID PRJNA1240274, which can be accessed using the link: https://www.ncbi.nlm.nih.gov/sra/PRJNA1240274. The raw qPCR data along with the sample metadata are available on Zenodo under the DOI: https://doi.org/10.5281/zenodo.14917324. The metaproteomics datasets generated during the current study are available in the PRIDE (PRoteomics IDEntifications Database) data repository with the sample metadata, vide PRIDE dataset identifier PXD062138, and this dataset can be accessed at https://www.ebi.ac.uk/pride.
