# Supplementary material for: Cover Crop Root Channels Promote Bacterial Adaptation to Drought in the Maize Rhizosphere
Source: Glob Chang Biol. 2025 Sep 20;31(9):e70512. doi: 10.1111/gcb.70512 (PMC12450045; doi:10.1111/gcb.70512)
Supplement: Supplementary file 1 — Figures S1‐S16: gcb70512‐sup‐0001‐Text‐FiguresS1‐S16.zip. [file GCB-31-e70512-s002.zip › gcb70512-sup-0001-FigureS1-S16@Supplemental_text_and_figures_S1-S16.docx]

Supplementary material

# Cover crop root channels promote bacterial adaptation to drought in the maize rhizosphere

Ghosh et al.

Correspondence: [nico.jehmlich@ufz.de](mailto:nico.jehmlich@ufz.de), [jochen.mueller@kit.edu](mailto:jochen.mueller@kit.edu)

## Methods

## Confirmatory factor analysis

We implemented a confirmatory factor analysis approach while designing the model, where we aimed at testing the maximum likelihood of data fit to the hypothesised equation pathway to interpret how physicochemical properties affect bacterial presence and role in the rhizosphere. We created a latent variable for the soil quality which included pH, bulk density and soil moisture content. Regressions with the latent variable were estimated with the soil content of total organic C and total N and the bacterial diversity. The CFA model equation looks like this:

*Latent variable:*

*Soil Quality =~ pH + Density + Moisture*

*Biological Activity =~ Community Diversity + Protein Diversity*

*Biological Activity ~~ Soil Quality*

*Regressions:*

*Carbon ~ Density + Soil Quality*

*Nitrogen ~ Density + Soil Quality*

*Community Diversity ~ Soil Quality*

The reason behind ´Soil Quality´ as a latent variable generated using measurements of soil bulk density, soil moisture contents and pH was considering the involvement of drought stress in this study that affects directly the soil bulk density and moisture content. Previous studies have reported decrease in moisture availability (Amtmann & Blatt, 2009) and soil bulk density (Zhang et al., 2019) with the formation of cracks, causing cavities in the soil and evaporating moisture. Soil pH was also included in this equation since we are investigating three soil types having different pH.

Alpha diversity indices can be used as a proxy tool for describing and comparing biodiversity (Finn, 2024), here in the different soil types under drought/rainfall-fed conditions and so we used it for both bacterial communities and proteins to compute ´Biological Activity´. Bacterial communities in the soil are influenced by the soil physicochemical properties (pH, soil bulk density and soil moisture content for soil quality), so we used covariances to identify the statistical correlation between them. Moisture is known to influence the pH of a system (Bell & Labuza, 1994) and we were also curious to know how the decreasing moisture content in the soil influenced the pH, henceforth the inclusion of a covariant relationship between pH and moisture. The presence of C and N are important variables inside soil profiles, and we used regressions to include them in our model to understand statistically how they influence the system.

The fitness of the equation model and the structural relationships with the data of the parameters were performed using the *lavaan* R package (v0.6-19) (Rosseel, 2012). The quality of the model was further verified using comparative fit index (CFI) (0.93_CFI_ > 0.9, which is the baseline for reasonable fit), standardised root-mean-square residual (SRMR) (anything < 0.8 is a good fit indicator) and low Akaike information criterion (AIC). The *lavaanplot* R package (v0.8.1) (Lishinski, 2024) helped us generate the graphical representation of the model, with the estimates and the significant associations.

## References

Amtmann, A., & Blatt, M. R. (2009). Regulation of macronutrient transport. New Phytologist, 181(1), 35-52. <https://doi.org/10.1111/j.1469-8137.2008.02666.x>

Bell, L. N., & Labuza, T. P. (1994). Influence of the low-moisture state on pH and its implication for reaction kinetics. *Journal of Food Engineering*, 22(1), 291-312. <https://doi.org/10.1016/0260-8774(94)90036-1>

Finn, D. R. (2024). A metagenomic alpha-diversity index for microbial functional biodiversity. FEMS Microbiology Ecology, 100(3). <https://doi.org/10.1093/femsec/fiae019>

Lishinski, A. (2024). *lavaanPlot: Path Diagrams for 'Lavaan' Models via 'DiagrammeR'. R package version 0.8.1*. GitHub. <https://github.com/alishinski/lavaanPlot>

Rosseel, Y. (2012). lavaan: An R Package for Structural Equation Modeling. *Journal of Statistical Software*, *48*(2), 1 - 36. <https://doi.org/10.18637/jss.v048.i02>

Zhang, Q., Shao, M., Jia, X., & Wei, X. (2019). Changes in soil physical and chemical properties after short drought stress in semi-humid forests. *Geoderma*, *338*, 170-177. <https://doi.org/10.1016/j.geoderma.2018.11.051>

## Supplementary Figures

Figure S1a: Soil moisture trends in the topsoil (0-30 cm) and subsoil (30-60 cm) along the experimental durations under drought (D) and rainfall-fed (RF) conditions in the Luvisol.

Figure S1b: Soil moisture trends in the topsoil (0-30 cm) and subsoil (30-60 and 60-120 cm) along the experimental durations under D and RF conditions in the Phaeozem.

 Figure S1c: Soil moisture trends in the topsoil (0-30 cm) and subsoil (30-60 and 60-120 cm) along the experimental durations under D and RF conditions in the Podzol.


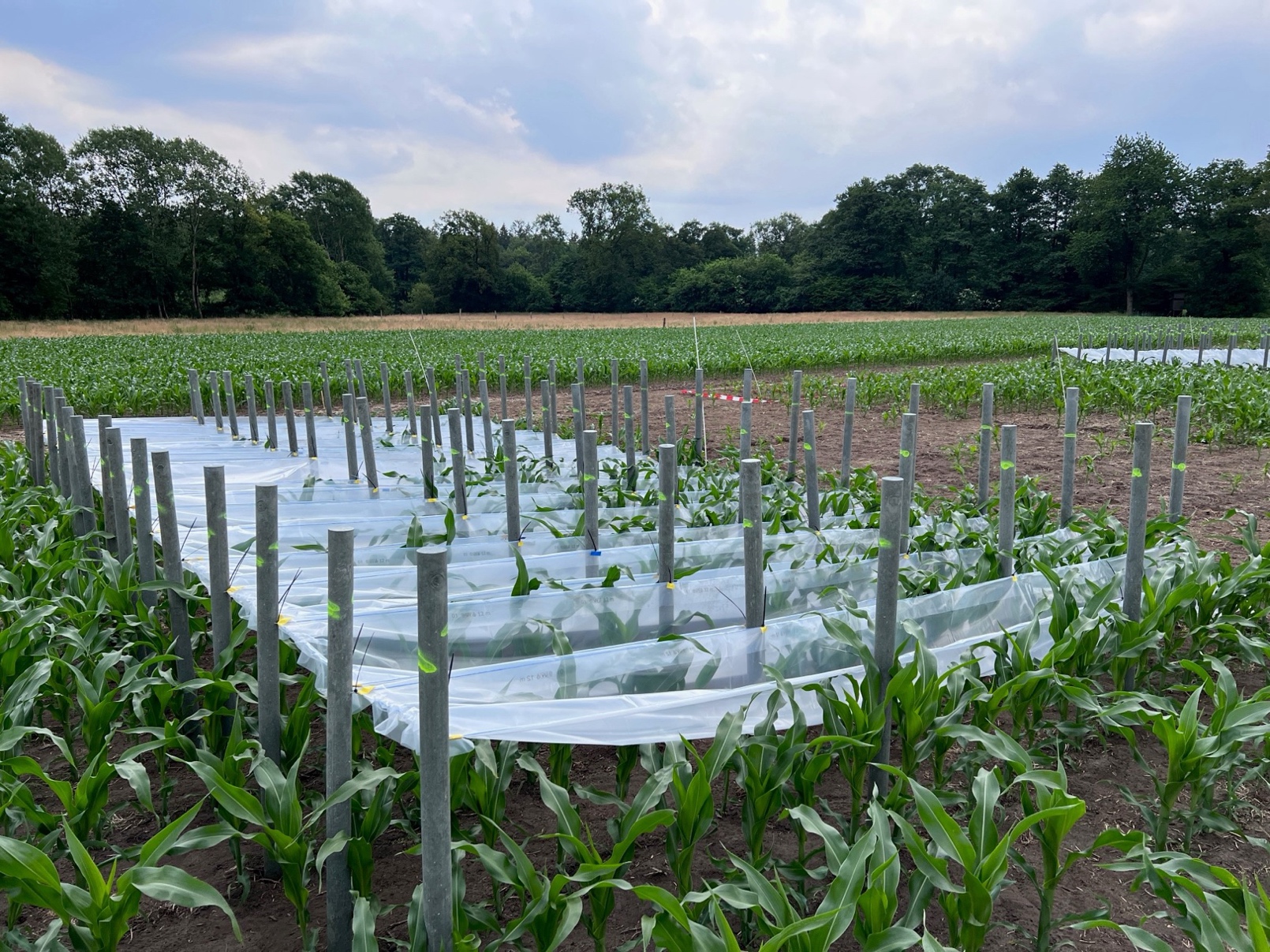


Figure S2: A pictorial representation of the rainout shelter setup for studying the impact of drought against rainfall-fed conditions for the strategy of reusing cover crop root channels in different soil types across Germany.

Figure S3: Pictures of the root overlap regions – the root channels of the winter cover crop regions reused by maize for deeper penetration into the soil profile. These were the regions from which the samples were collected for the ‘maize root in cover crop root channels (MCR)’ samples.

Figure S4: A bar-chart representing the different values of the physicochemical properties that have been measured at the three soil sampling sites with different soil types – Luvisol (Hohenschulen), Phaeozem (Reinshof) and Podzol (Karkendamm). All values are provided in Table S2.

Figure S5: An alluvial flowchart representing the different variations and the identified bacterial phyla in each one of them at the three soil sampling sites.

Figure S6: The conceptual confirmatory factor analysis (CFA) model evaluating the probable relationships between the soil physicochemical properties with the parameters of biological activity (bacterial and protein diversity). The numbers next to the edges are estimates of the weightage of the relationships between the parameters. The quality of the model was further verified using comparative fit index (CFI) (0.93_CFI_ > 0.9, which is the baseline for reasonable fit), standardised root-mean-square residual (SRMR) (0.07 < 0.8, which is a good fit indicator) and low Akaike information criterion (AIC). The asterisks next to the arrows indicate significance of the interaction between the two parameters; **p* < 0.05, ****p* < 0.001. A summary of the analysis can be found in Table S13.

Figure S7: A heat-tree representation of the differential bacterial abundance for the cover crop variations and fallow conditions. The *log2* values of the relative bacterial abundance between treatments and fallow are shown by two colours, where ‘pink4’ corresponds to the name of the variation at the top and ‘lightgreen’ to the name on the right side (RStudio colour palettes have been used for colour representation).

Figure S8: A faceted boxplot representation of a correlation between the soil physicochemical properties (pH, soil moisture content, bulk density, total organic carbon (TOC), and total nitrogen (TN)) plus bacterial community richness with the cover crop variations (where Fallow is the control and Legume/Grass and Brassica/Grass are treatments). Significance among the cover crop variations being used are represented by compact letter display (CLD) representations.

Figure S9: A heat-tree representation of the differential bacterial abundance in the two layers of soil depth – topsoil and subsoil. The *log2* values of the relative bacterial abundance between the layers are shown by two colours, where ‘lightorange’ corresponds to topsoil and ‘lightblue4’ to subsoil (RStudio colour palettes have been used for colour representation).

Figure S10: A faceted boxplot representation of a correlation between the soil physicochemical properties (pH, soil moisture content, bulk density, total organic carbon (TOC), and total nitrogen (TN)) plus bacterial community richness with the soil sampling depth of topsoil and subsoil. Significance among the cover crop variations being used are represented by compact letter display (CLD) representations.

Figure S11: A summary of the expression of enzymes involved in amino acid synthesis and degradation and proline metabolism under drought and rainfall-fed conditions in our different sampling sites and the different cover crop variations.

Figure S12: A summary of the differential expression of proteins of these pathways at the three sampling sites in the topsoil and the subsoil, after the re-use of the cover crop root channels. Here, the Log2FC values of the treatments (Legume/Grass and Brassica/Grass) have been deducted by the values from fallow to see the net change in the expression of proteins belonging to these pathways.

Figure attached as a vector file (Supplementary Figure S13, PDF)

Figure S13: The extended heatmap from metaproteomics, which includes all the enzymes heavily or sparsely involved in the different functional cycles and bacterial phyla corresponding to the enzymes in the Luvisol. For proteomics, *n* = 39. (Additional vector file provided for detailed observations)

Figure attached as a vector file (Supplementary Figure S14, PDF)

Figure S14: The extended heatmap from metaproteomics, which includes all the enzymes heavily or sparsely involved in the different functional cycles and bacterial phyla corresponding to the enzymes in the Phaeozem. For proteomics, *n* = 40. (Additional vector file provided for detailed observations)

Figure attached as a vector file (Supplementary Figure S15, PDF)

Figure S15: The extended heatmap from metaproteomics, which includes all the enzymes heavily or sparsely involved in the different functional cycles and bacterial phyla corresponding to the enzymes in the Podzol. For proteomics, *n* = 40. (Additional vector file provided for detailed observations)

Figure S16: A summary of cellulase expression under drought and rainfall-fed conditions in our different sampling sites and the different cover crop variations. The identified cellulases were mapped to bacterial communities in order to find out which phylum plays a major role in breaking down cellulose.
