# Supplementary figures and images for: Cover Crop Root Channels Promote Bacterial Adaptation to Drought in the Maize Rhizosphere
Source: Glob Chang Biol. 2025 Sep 20;31(9):e70512. doi: 10.1111/gcb.70512 (PMC12450045; doi:10.1111/gcb.70512)

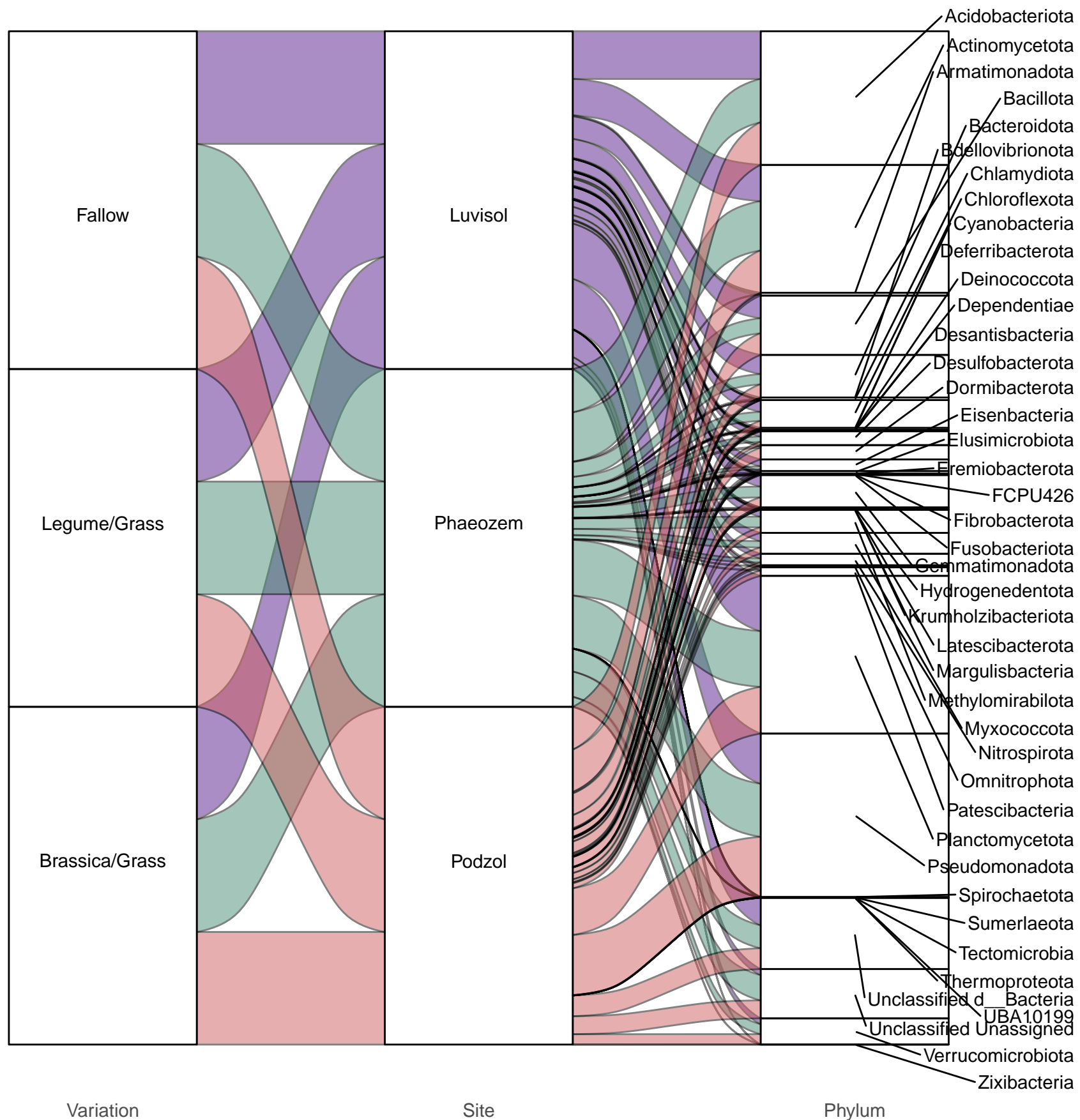

Site Luvisol Phaeozem Podzol

Supplement: Supplementary file 1 — Figures S1‐S16: gcb70512‐sup‐0001‐Text‐FiguresS1‐S16.zip. [file GCB-31-e70512-s002.zip › gcb70512-sup-0002-FigureS5@Figure_S5.pdf]

Log<sub>2</sub> FC (Drought/Rainfall-fed)

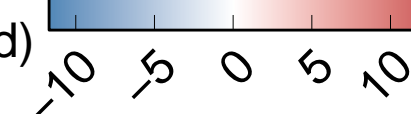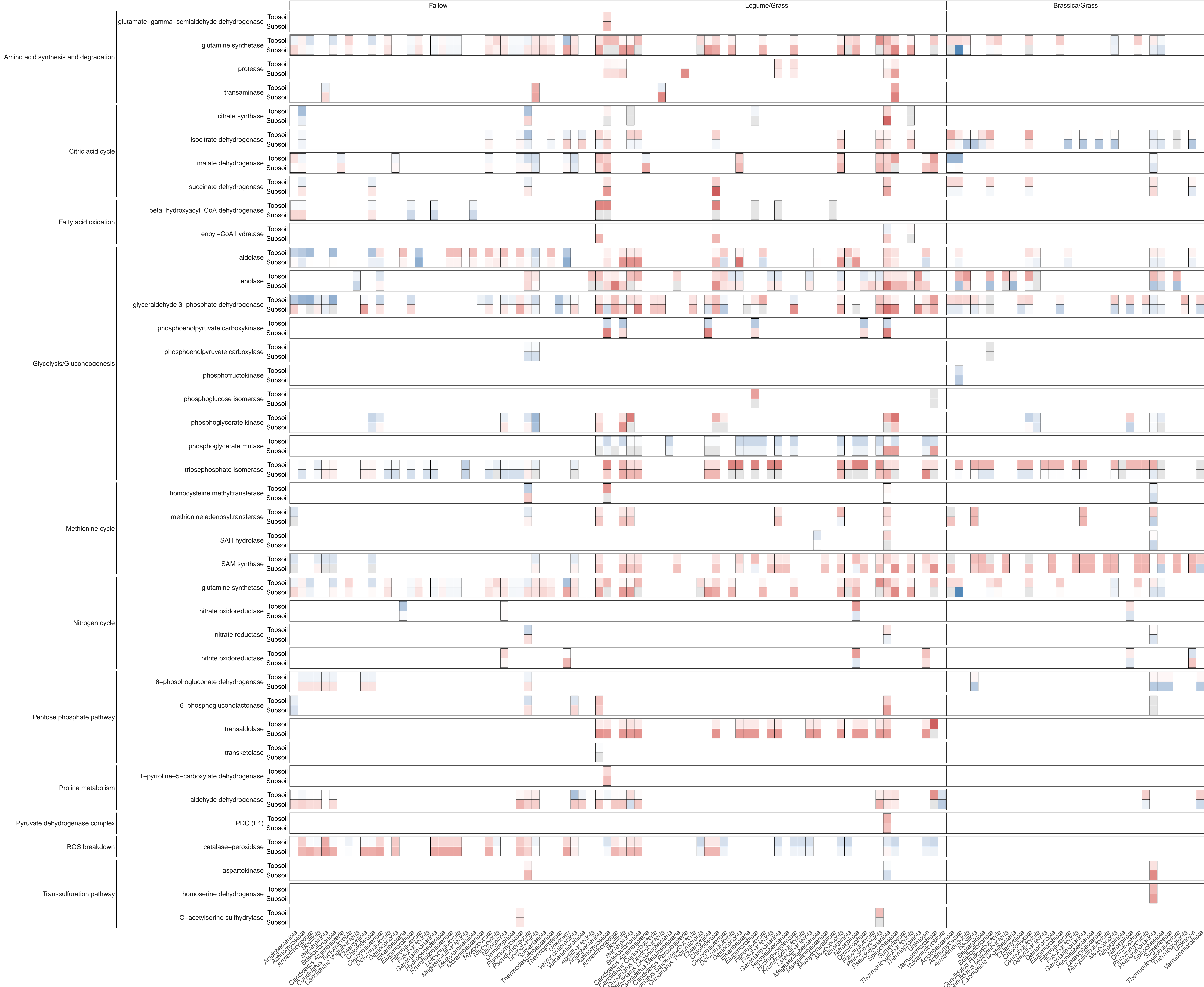

Supplement: Supplementary file 1 — Figures S1‐S16: gcb70512‐sup‐0001‐Text‐FiguresS1‐S16.zip. [file GCB-31-e70512-s002.zip › gcb70512-sup-0003-FigureS13@Figure_S13.pdf]

Log<sub>2</sub> FC (Drought/Rainfall-fed)

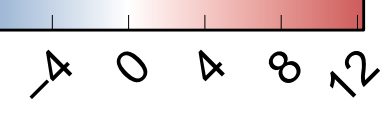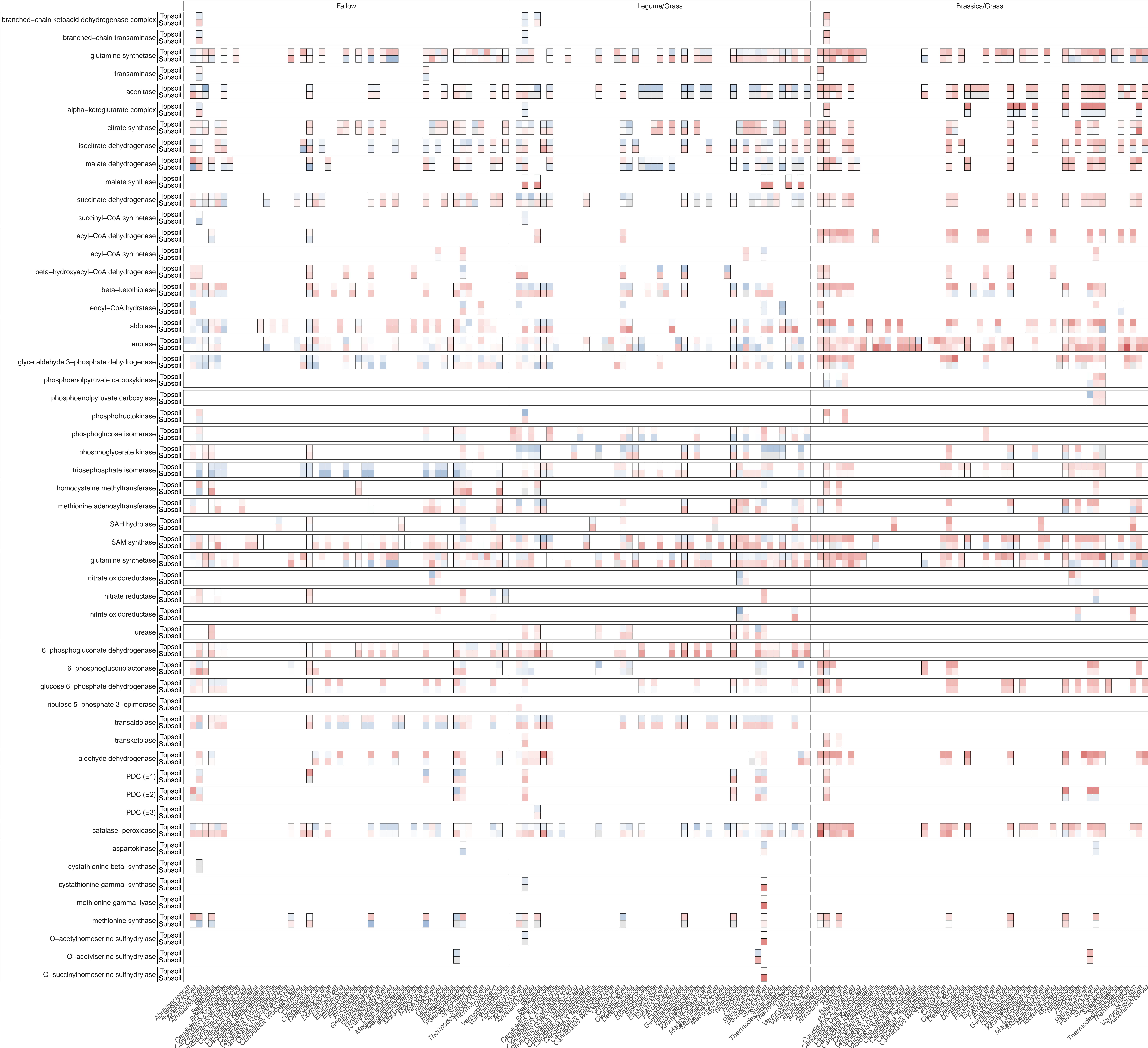

Supplement: Supplementary file 1 — Figures S1‐S16: gcb70512‐sup‐0001‐Text‐FiguresS1‐S16.zip. [file GCB-31-e70512-s002.zip › gcb70512-sup-0004-FigureS14@Figure_S14.pdf]

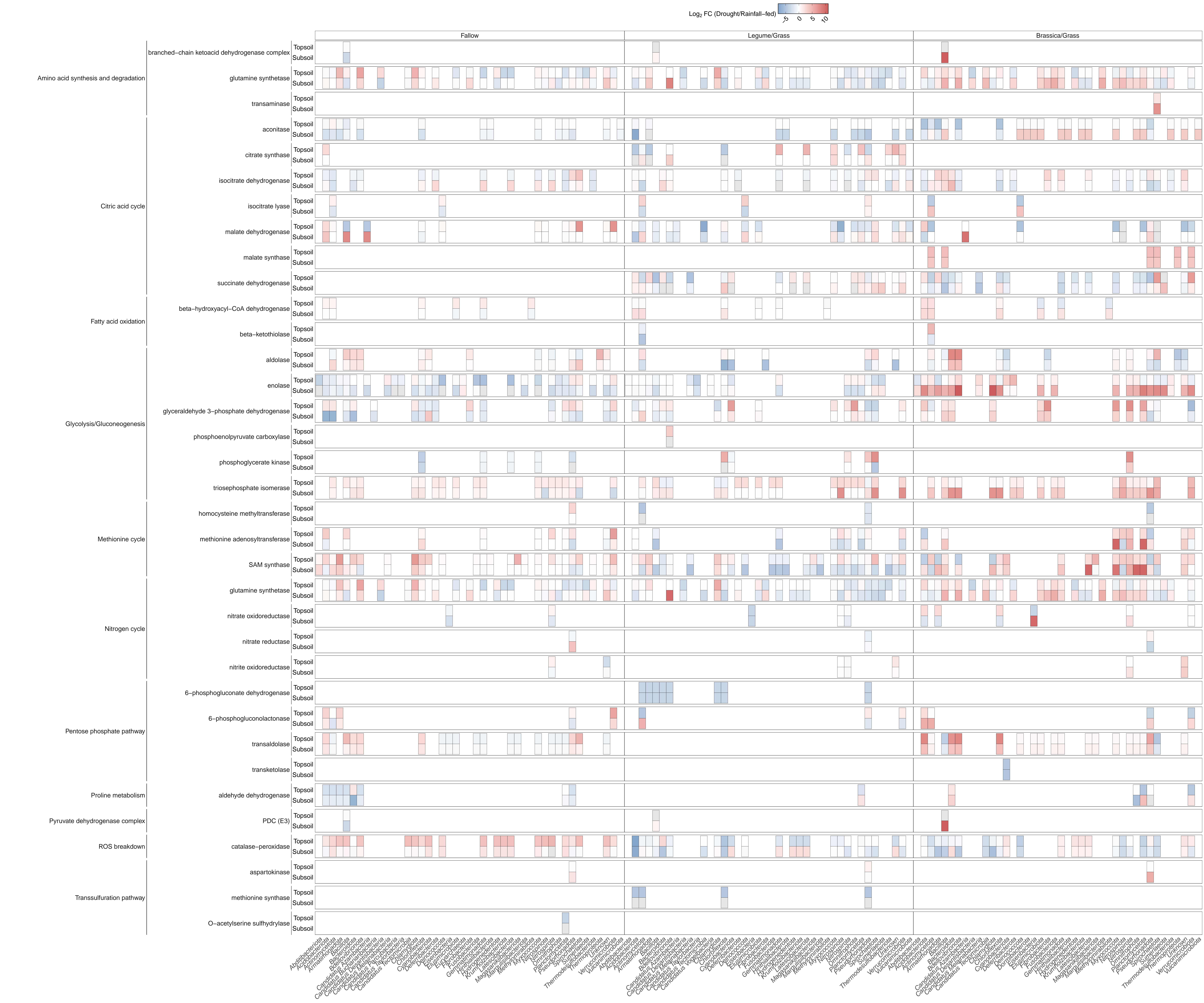

Supplement: Supplementary file 1 — Figures S1‐S16: gcb70512‐sup‐0001‐Text‐FiguresS1‐S16.zip. [file GCB-31-e70512-s002.zip › gcb70512-sup-0005-FigureS15@Figure_S15.pdf]
